# Analysis of HrpG regulons and HrpG‐interacting proteins by ChIP‐seq and affinity proteomics in *Xanthomonas campestris*

**DOI:** 10.1111/mpp.12903

**Published:** 2020-01-08

**Authors:** Hong‐Yu Zhang, Jin‐Wei Wei, Wei Qian, Chao‐Ying Deng

**Affiliations:** ^1^ State Key Laboratory of Plant Genomics Institute of Microbiology Chinese Academy of Sciences Beijing China; ^2^ College of Life Sciences University of Chinese Academy of Sciences Beijing China

**Keywords:** ChIP, HrpG, regulon, TAP, virulence, *Xanthomonas campestris*

## Abstract

Gamma‐proteobacteria *Xanthomonas* spp. cause at least 350 different plant diseases among important agricultural crops, which result in serious yield losses. *Xanthomonas* spp. rely mainly on the type III secretion system (T3SS) to infect their hosts and induce a hypersensitive response in nonhosts. HrpG, the master regulator of the T3SS, plays the dominant role in bacterial virulence. In this study, we used chromatin immunoprecipitation followed by sequencing (ChIP‐seq) and tandem affinity purification (TAP) to systematically characterize the HrpG regulon and HrpG interacting proteins in vivo. We obtained 186 candidate HrpG downstream genes from the ChIP‐seq analysis, which represented the genomic‐wide regulon spectrum. A consensus HrpG‐binding motif was obtained and three T3SS genes, *hpa2*, *hrcU*, and *hrpE*, were confirmed to be directly transcriptionally activated by HrpG in the inducing medium. A total of 273 putative HrpG interacting proteins were identified from the TAP data and the DNA‐binding histone‐like HU protein of *Xanthomonas campestris* pv. *campestris* (HU_xcc_) was proved to be involved in bacterial virulence by increasing the complexity and intelligence of the bacterial signalling pathways in the T3SS.

## Graphical abstract

HrpG, a global regulator, plays the central role in bacterial virulence and physiological pathways.

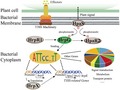

## 1 INTRODUCTION

To optimize the living environment in host cells, gram‐negative bacterial pathogens rely mainly on the type III secretion system (T3SS) to manipulate a variety of essential biological processes (Dodds and Rathjen, 2010; Dey *et al*., 2019). Characterizing the spatiotemporal expression patterns, the assembled structure, and the secreted/translocated components of the T3SS is therefore critical. In phytopathogenic bacteria the T3SS components are encoded by a large conserved cluster of genes that includes *hrp* (hypersensitive response [HR] and pathogenicity), *hrc* (*hrp* conserved), and *hpa* (*hrp* associated) (Kim, *et al.*, 2003; Li, *et al.*, 2011). These genes are controlled by several transcription factors, including HrpG, which is the master regulator of T3SS genes. HrpG contains an N‐terminal response regulator receiver domain and a C‐terminal DNA‐binding motif. It is an OmpR (outer membrane porin regulator) family response regulator of the two‐component signal transduction system and the aspartic acid residue in its response regulator receiver domain is a conserved phosphorylation site (Wengelnik and Bonas, 1996; Wengelnik *et al.*, 1996). HrpG is at the top of the regulatory cascade that regulates the expression of T3SS genes with or without intermediately regulating the AraC‐type (arabinose catabolism) regulator HrpX. Therefore, HrpG plays the central role in bacterial virulence, which causes disease and pathogenicity in susceptible hosts and elicits HR (Willis *et al.*, 1991; Lindgren, 1997). Deletion of *hrpG* abolished the expression of T3SS genes and reduced the bacterial virulence (Zhang *et al.*, 2018). Moreover, the expression levels of *hrpG* and the T3SS genes were repressed in complex or rich media, such as nutrient yeast glycerol (NYG) medium, but specifically induced in planta and in minimal media, such as XVM2 and XCM2 (Wengelnik *et al.*, 1999).

Together with the rapid development of biochemical technologies and comparative genomics, remarkable progress has been made in understanding the role of HrpG, mainly in *Xanthomonas* spp. The gamma‐proteobacteria *Xanthomonas* spp. cause at least 350 different plant diseases in important agricultural crops such as rice, tomato, citrus, cassava, sugar cane, and brassica. Disease symptoms include wilting, necrosis, cankers, spots, and blight in plant leaves, stems, and fruits, resulting in serious yield losses. The *hrpG* gene was first identified in *X. campestris* pv. *vesicatoria*, which is the causal agent of bacterial spot disease of pepper and tomato (Wengelnik *et al.*, 1996, 1999). Subsequently, genes involved in transcriptional activation of *hrpG*, such as *trh* and *gamR* (Tsuge *et al.*, 2006; Rashid *et al.*, 2016), transcriptional repression of *hrpG*, such as *lrpX* (Islam *et al.*, 2009), transcript stability of *hrpG*, such as *rsmA* (Andrade *et al.*, 2014), genes regulated by HrpG, such as *hpaR*, *pghAxc*, and *pghBxc* (Wei *et al.*, 2007; Wang *et al.*, 2008), and putative cognate sensor kinase for HrpG (Li *et al.*, 2014), were identified in *X. oryzae* pv. *oryzae* and *X. campestris* pv. *campestris* (Xcc), which are the causal agents of bacterial leaf blight of rice and black rot disease of cruciferous crops.

In addition, “omics”‐based screenings of the HrpG regulon have been performed using, for example, cDNA amplified restriction fragment polymorphism (cDNA‐AFLP) to analyse gene transcriptional expression changes. Thirty *hrpG*‐induced and five *hrpG*‐repressed cDNA fragments were identified by cDNA‐AFLP and defined as the *hrpG* regulon in *X. campestris* pv. *vesicatoria*. The expression of most of the *hrpG* regulon genes was dependent on *hrpX* (Noel *et al*., 2001). Subsequently, DNA microarray analysis revealed 232 and 181 genes that belonged to the HrpG and HrpX regulons, respectively; 123 of these genes overlapped with genes in *X. axonopodis* pv. *citri* (Xac; Guo *et al.*, 2011). This was the first robust and comprehensive whole‐genome study of the HrpG regulon. A transcriptomic analysis of the *hrpG* regulon in an ectopically expressing *hrpG* mutant of *X. campestris* pv. *raphani* by RNA sequencing detected 134 induced and 7 repressed genes (Roux *et al.*, 2015). These three techniques indirectly characterized the transcriptional expression of genes influenced by *hrpG*. In recent years, chromatin immunoprecipitation followed by sequencing (ChIP‐seq) has been used widely for the global mapping of transcription factor binding sites to determine directly regulated genes. Promoter regions that co‐immunoprecipitate with the transcription factors are enriched in the ChIP‐seq data (Galagan *et al.*, 2013).

To date, specifying genes that are directly regulated by HrpG has been a major challenge (Büttner and Bonas, 2010; Bai *et al.*, 2018). Moreover, the interacting proteins involved in HrpG activity and function also remains to be confirmed. In the present study, we performed ChIP‐seq to systematically detect the promoter regions of genes directly bound by HrpG in vivo. We obtained 186 candidate HrpG downstream genes and the consensus HrpG‐binding motif. In vitro electrophoretic mobility shift assay (EMSA) and in vivo real‐time quantitative reverse‐transcription PCR (RT‐qPCR) revealed three T3SS genes, *hpa2*, *hrcU*, and *hrpE*, whose transcription was directly activated by HrpG in the inducing medium and thus participated in bacterial virulence. In vivo tandem affinity purification (TAP) together with mass spectrometry techniques revealed 273 putative HrpG‐interacting proteins under induction. Among these, HrpG physically interacted with the DNA‐binding histone‐like HU protein of Xcc (XC3262, HU_xcc_), which is also involved in Xcc virulence. This finding indicates the complexity of the bacterial signalling pathways in the T3SS.

## 2 RESULTS

### 2.1 Genome‐wide analysis of the HrpG regulon by ChIP‐seq

To detect genes that are regulated directly by HrpG (XC3077) in Xcc strain 8004, we identified HrpG‐binding promoters and dissected the HrpG regulon by ChIP‐seq. A recombinant bacterial strain (ΔhrpG‐hrpG‐his_6_) was constructed in which full‐length HrpG was fused with a C‐terminal His_6_ tag and overexpressed in trans in the broad‐host vector pHM1 in the genetic background of the *hrpG* mutant. This strain phenocopied the wild‐type (WT) strain in bacterial virulence against the host cabbage (*Brassica oleraceae* ‘Jingfeng No. 1’) and HR (*Nicotiana tabacum* ‘SR1’) (Figure S1a‐c), indicating that the C‐terminal His_6_ tag had no remarkable impact on HrpG function. Western blotting also confirmed that HrpG‐His_6_ was expressed in vivo and immunoprecipitated by His_6_ monoclonal antibody (Figure S1d).

**Figure 1:**
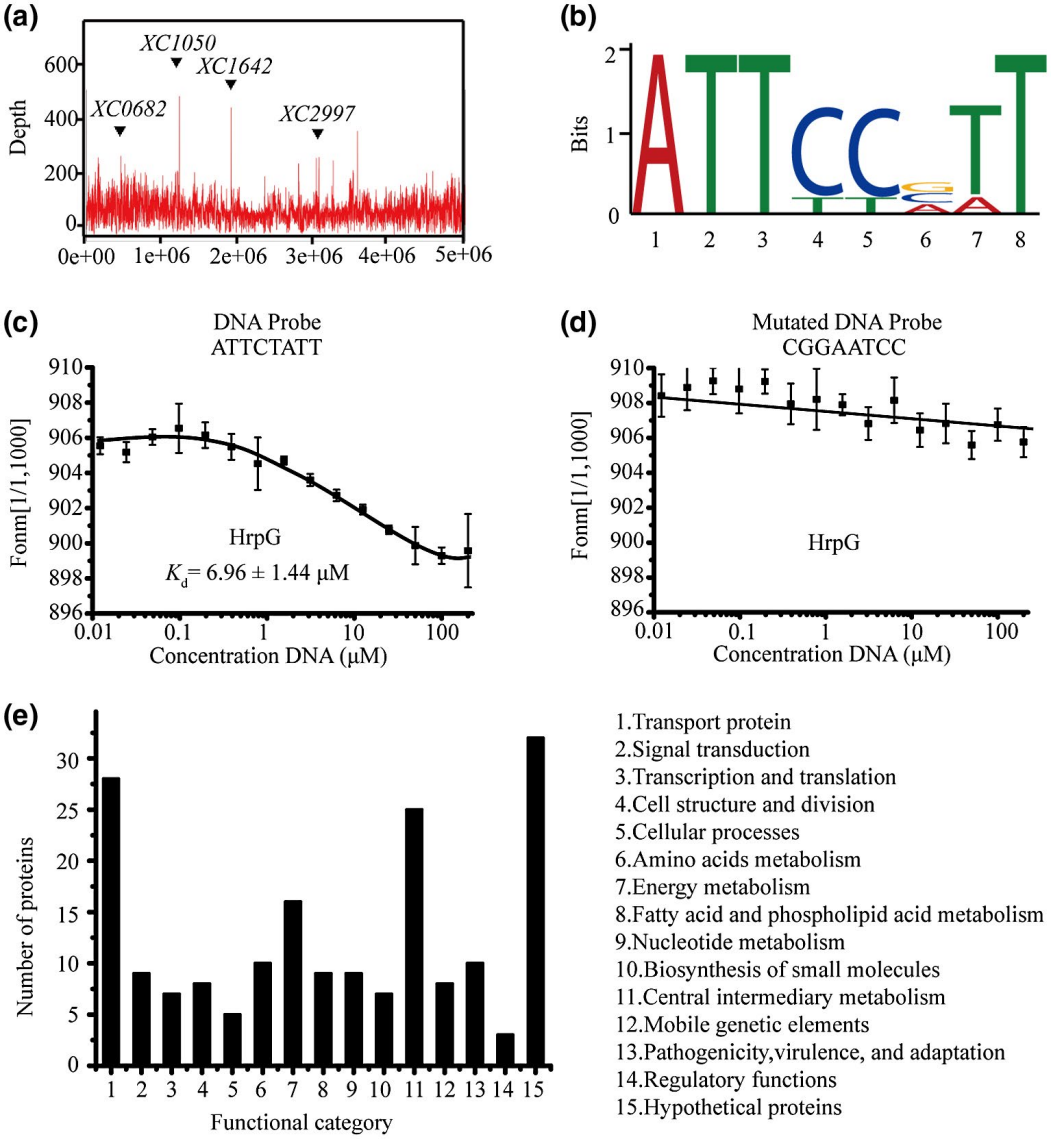
Genome‐wide analysis of the *hrpG* regulon by ChIP‐seq in the XCM2 inducing medium. (a) Peak calling of *hrpG*. The *hrpG*‐bound landscape in *Xanthomonas campestris* pv. *campestris* (Xcc) strain 8004. (b) Predicted consensus *hrpG*‐binding DNA motifs based on the ChIP‐seq data and MEME analysis. WebLogo was used to visualize the nucleotide composition; the height of each nucleotide is proportional to its conservation at that site. (c), (d) Verification of *hrpG*‐binding DNA motif by microscale thermophoresis (MST) analysis. Two oligonucleotides were synthesized and incubated with the labelled HrpG protein in an Nano Temper standard capillary in the MST assay. The MST analysis was used to measure the interaction between the HrpG protein and the oligonucleotide (c) wild‐type and (d) mutated DNA probes. The sequences of the oligonucleotides are listed in Table S2. The solid curve is the fit of the data points to the standard KD‐Fit function. The black bars indicate standard deviations (*n* = 3). *K*_d_ is the dissociation constant. (e) Functional categories of the genes with promoters that putatively bound by HrpG. Details of the genes are given in Table S3

Previous studies suggested that *hrp* expression in *Xanthomonas* was induced in minimal media and repressed in rich media (Schulte and Bonas, 1992a,b), and showed that XCM2 was one of the most effective inducing media (Jiang *et al.*, 2013). Therefore, we selected the XCM2 medium as the inducing condition for the HrpG regulon of Xcc. The recombinant bacterial strain (ΔhrpG‐hrpG‐his_6_) was cultured in NYG medium until the OD_600_ was 0.4 ± 0.05, and then cultured in XCM2 medium for 2 hr. After induction, total bacterial proteins were collected by extraction. The DNA fragments that co‐immunoprecipitated with the HrpG‐His_6_ protein using an anti‐His_6_ monoclonal antibody were enriched and purified. Subsequent high‐throughput sequencing and peak calling of the ChIP‐seq data (Figure 1a) identified 186 sites in the 5′ promoter regions (<300 bp) of genes (Table S3), suggesting that HrpG directly bound to the promoters to modulate the transcription of the corresponding genes. The ChIP‐seq data and MEME analysis (Bailey *et al.*, 2015) of these promoters were used to predict the consensus HrpG‐binding motif (Figure 1b). To verify this motif experimentally, a 5′‐FAM (carboxyfluorescein)‐labelled oligonucleotide was synthesized based on the consensus sequence and used as the DNA probe in the microscale thermophoresis (MST) analysis, which is a powerful technique to quantify biomolecular interactions. It revealed that the purified HrpG protein physically bound to the 5′‐FAM oligonucleotides in vitro, with a dissociation constant (*K*_d_) of 6.96 ± 1.44 μM (Figure 1c), and that binding was eliminated when the conserved nucleotides of the probe were mutated, as shown in Figure 1d. These results confirmed the presence of the HrpG‐binding motif in the promoters of Xcc genes [ATT(C/T)(C/T)(G/C/A)(T/A)T)]. This motif has not been reported previously.

The putative HrpG‐regulated genes were assigned to 15 functional categories according to the gene ontology (GO) annotations (The Gene Ontology, 2019) and KEGG pathways (Kyoto Encyclopedia of Genes and Genomes, https://www.kegg.jp/kegg/pathway.html) (Figure 1e). Besides the genes related to pathogenicity, virulence, and adaptation, HrpG also bound to the promoter regions of genes involved in transportation, signal transduction, transcription, and translation. This indicated that HrpG was not only a critical regulator of T3SS gene expression, but also was involved in other physiological pathways and coordinated bacterial function much more widely. The ChIP‐seq analysis identified a two‐component system sensor histidine kinase (VgrS; XC1050), which is essential in regulating bacterial virulence and multiple stress responses (Wang *et al.*, 2016), and eight other two‐component signalling system proteins, suggesting that HrpG may be involved in monitoring environmental and intracellular stimuli by directly regulating the expression of two‐component signalling system genes. Notably, a gene that encodes a type VI secretion protein (XC0699) also was identified in the ChIP‐seq data. The type VI secretion system (T6SS) is a bacterial molecular mechanism that injects toxic effectors into host cells and thus is involved in bacterial–host competition and interaction (Ho *et al.*, 2014; Bernal *et al.*, 2018). Like the T3SS, the T6SS also is widespread among gram‐negative bacteria. The identification of a type VI secretion protein in our ChIP‐seq data suggested that the putative crosstalk between T3SS and T6SS needed further investigation. Although the electrophoretic mobility shift assay (EMSA) verified the physical binding between HrpG and the promoter of the well‐documented downstream gene *hrpX* (Figure S2b), *hrpX* was not detected by ChIP‐seq. This may be because of the low abundance of *hrpX* promoters, leading to low amplification with the adaptor primers.

### 2.2 HrpG regulates the expression of downstream genes by physically binding to their promoters

**Figure 2:**
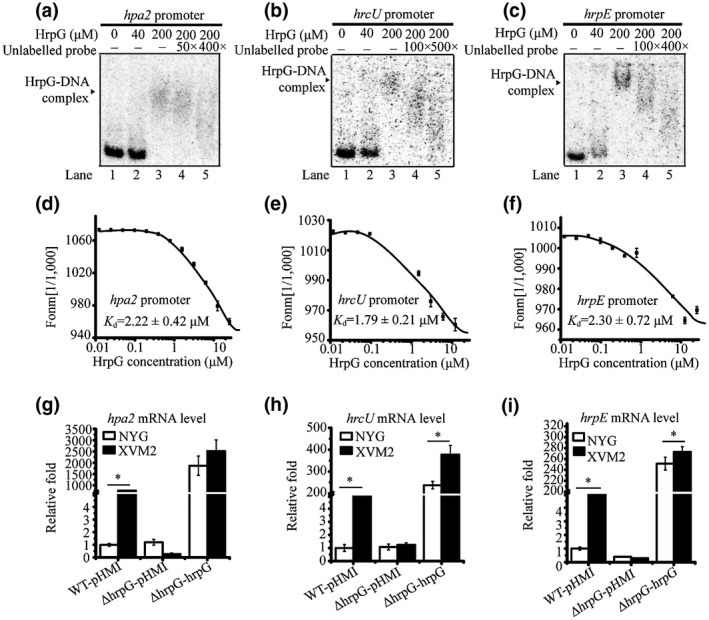
The *hrpG* gene regulates the expression of downstream genes by directly binding to their promoters. (a)–(c) Electrophoretic mobility shift assay (EMSA) revealed that HrpG directly bound the promoter region of downstream genes. PCR products of the promoter regions of *hpa2* (XC3001), *hrcU* (XC3012), and *hrpE* (XC3021) were labelled with [γ‐^32^P]ATP and used as DNA probes. Increasing numbers of unlabelled DNA probes were used as competitors. Each experiment was repeated three times. Triangles indicate HrpG–DNA complexes. (d)–(f) Verification of *hrpG* binding to the promoter regions of downstream genes by microscale thermophoresis (MST) analysis. The 5′‐FAM (carboxyfluorescein)‐labelled oligonucleotide primers were synthesized and annealed with an unlabelled complementary primer to form double‐stranded (ds) DNA. The labelled dsDNA was added to serially diluted HrpG protein in a Nano Temper standard capillary in the MST assay. The solid curve is the fit of the data points to the standard KD‐Fit function. The black bars indicate standard deviations (*n* = 3). *K*_d_ is the dissociation constant. (g)–(i) HrpG positively controls the transcription of *hpa2*, *hrcU*, and *hrpE*. Quantitative reverse transcription PCRs (RT‐qPCRs) were performed to quantify the *hrpG* mRNA levels in different bacterial strains before and after induction in XVM2 medium. Amplification of the cDNA of 16S rRNA was used as an internal control. Each experiment was completed with three biological replicates. A representative result of three independent experiments is shown. Vertical bars indicate the standard deviation (*n* = 3). Asterisks indicate significant differences under the non‐inducing (rich medium, NYG) and inducing medium (XVM2) culture conditions (Student's *t* test, *p* ≤ .05)

To preliminary screen the HrpG‐regulated genes, the in vitro biotin‐labelled EMSA was used to confirm the physical binding of representative genes. We selected 16 candidate genes from the ChIP‐seq data and 12 promoter probes that competed with increasing amounts of HrpG to detect possible binding events in vitro (Figure S2a). Furthermore, we optimized the [γ‐^32^P]ATP‐labelled EMSA using the promoter of *hrpX* (*hrpXp*) and *hrpA* operon (*hrpAp*)–HrpG binding as a positive control (Figure S2b) (Wengelnik, 1996; Wengelnik *et al.*, 1999; Ficarra *et al.*, 2015). The EMSA verified physical binding between HrpG and *hpa2p* (a lytic transglycosylase), *hrcUp* (an EscU/YscU/HrcU family T3SS export apparatus switch protein), *hrpEp* (HPr kinase) (Figure 2a‐c), as well as *vgrSp* (sensor histidine kinase) and the promoters of TonB‐dependent receptor (XC0124), pectin methylesterase (XC0125), and pectate lyase (XC1298) (Figure S2b). When increasing numbers of unlabelled probes were added to the EMSA reaction mixtures as competitors, the isotopic signals representing HrpG–DNA complexes gradually decreased. In addition, the MST analysis using 5′‐FAM‐labelled promoter fragments produced equilibrium binding constants of 2.22 ± 0.42 μM, 1.79 ± 0.21 μM, and 2.30 ± 0.72 μM for the HrpG–*hpa2p*, HrpG–*hrcUp*, and HrpG–*hrpEp* interactions, respectively (Figure 2d‐f), which suggested relatively strong binding affinities and protein–DNA interactions.

To determine whether the transcript levels of the seven genes that were verified to bind to HrpG were under the direct control of HrpG, RT‐qPCRs were performed in different Xcc strains. When WT Xcc was transferred from the rich NYG medium to the T3SS‐inducing XVM2 medium, the transcript levels of *hpa2*, *hrcU*, and *hrpE* increased by 764.2‐, 42.6‐, and 91.0‐fold, respectively (Figure 2g‐i). This result is consistent with the existing concept that the expression of the *hrp‐hrc‐hpa* T3SS genes generally is suppressed in rich media but induced in nutrition‐limited media that mimic the conditions in the host plant (Schulte and Bonas, 1992b; Wei *et al.*, 2000; Xiao *et al.*, 2007). The high induction of these genes was nearly abolished in the *hrpG* mutant strain (∆hrpG‐pHM1), whereas genetic complementation by overexpressing *hrpG* fully restored the expression of the downstream genes (Figure 2g‐i). In addition, when Xcc was grown in the inducing XVM2 medium, the *hpa2*, *hrcU*, and *hrpE* mRNA levels in the *hrpG* mutant decreased by 764‐, 41.4‐, and 91‐fold, respectively, compared with the WT, and genetic complementation of *hrpG* (∆hrpG‐hrpG) restored the expression levels to the WT levels (Figure 2g‐i). These experiments confirmed that HrpG directly and positively regulated *hpa2*, *hrcU*, and *hrpE* at the transcription level in the inducing medium. These results support the previous findings that *hpa2*, *hrcU*, and *hrpE* are clustered *hrp* genes that are regulated by *hrpX* (Zou, 2006), indicating that delicate regulatory crosstalk may take place between HrpX and HrpG.

**Figure 3:**
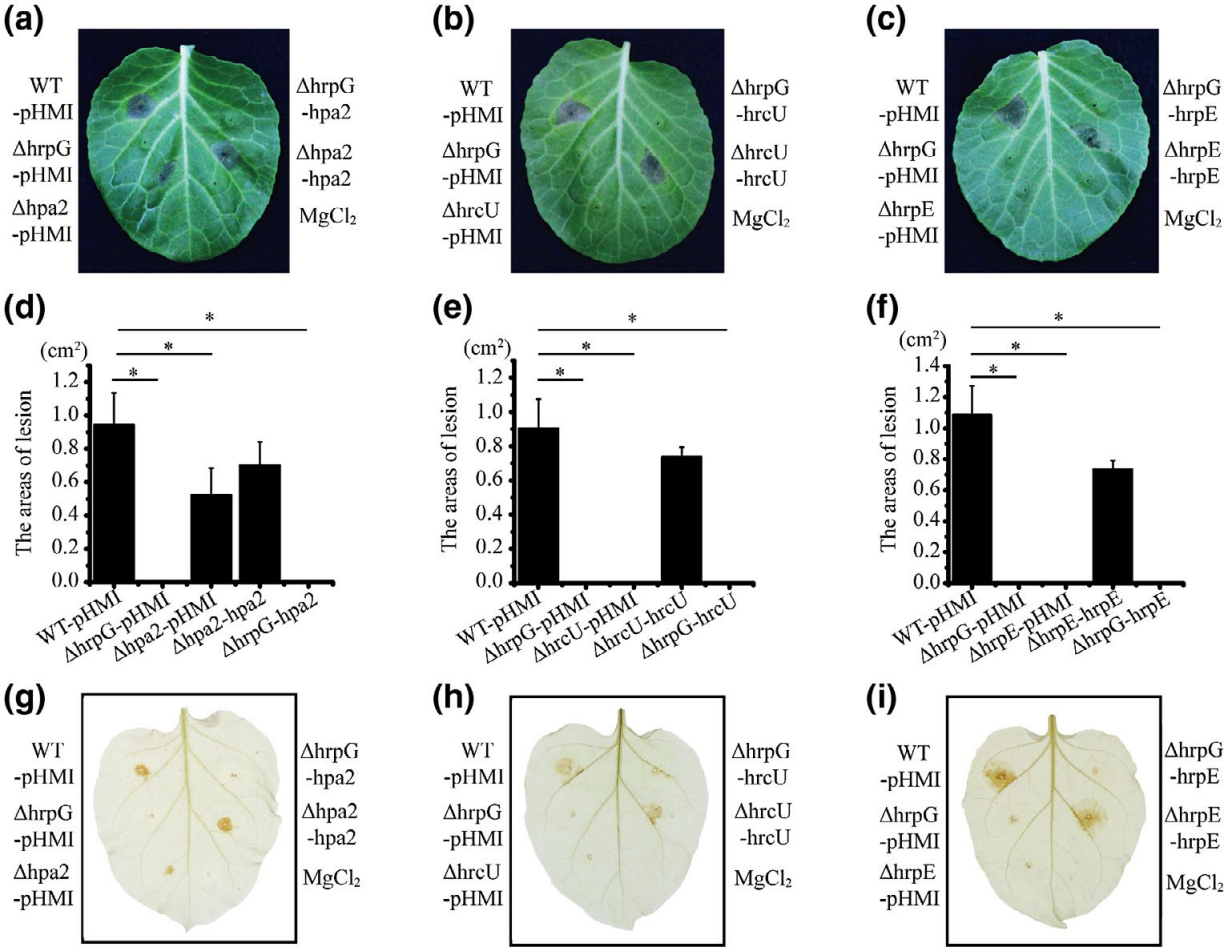
The *hpa2*, *hrcU*, and *hrpE* phenotypes involved in the type III secretion system (T3SS). (a)–(c) Virulence assay of *Xanthomonas campestris* pv. *campestris* (Xcc) strains against host plant (*Brassica oleracea* ‘Jingfeng No. 1’). Bacterial strains were inoculated onto the host cabbage, with 10 mM MgCl_2_ used as negative control. (d)–(f) The virulence level of the bacterial strains was measured. The areas of lesion were recorded 5 days after inoculation. Vertical bars indicate the standard deviation (*n* = 6). Asterisks indicate significant differences compared with the wild‐type (WT) strain (Student's *t* test, *p* ≤ .05). (g)–(i) Hypersensitive response (HR) assay of bacterial strains against nonhost plant (*Nicotiana tabacum* ‘SR1’). The bacteria lost nearly all their ability to produce a HR. *hpa2*, *hrcU*, and *hrpE* were overexpressed in the *hrpG* mutant genetic background. Bacterial strains were inoculated onto nonhost tobacco, with 10 mM MgCl_2_ used as a negative control

To verify that the three HrpG‐regulated downstream genes contribute to the phenotypic deficiencies of the HrpG mutant, we conducted mutational analyses by separately overexpressing *hpa2*, *hrcU*, and *hrpE* in each mutant or in the *hrpG* mutant background using a recombinant pHM1 vector. The virulence level in susceptible host cabbage (*B. oleracea* ‘Jingfeng No. 1’) and HR (*N. tabacum* ‘SR1’) were used to characterize the phenotypes. HrpG is the master regulator of T3SS, so deletion of *hrpG* is expected to influence the global virulence regulation and nutrient and energy metabolism (Mole *et al.*, 2007). We therefore speculated that deletion of downstream T3SS genes would impair the virulence of Xcc and that genetic complementation of only one regulated gene would not fully restore the detrimental phenotype. The *hpa2*, *hrcU*, and *hrpE* in‐frame deletion mutants (∆hpa2, ∆hrcU, and ∆hrpE) had significantly decreased virulence in the host plant (Figure 3a‐f) and lower HR (Figure 3g‐i), and genetic complementation restored the deficiency phenotype to levels similar to that of the WT (Figure 3). Moreover, overexpression of *hpa2*, *hrcU*, or *hrpE* in the *hrpG* deletion background (∆hrpG‐hpa2, ∆hrpG‐hrcU, ∆hrpG‐hrpE) only weakly restored the *hrpG* virulence deficiency phenotype (Figure 3).

### 2.3 Detecting HrpG binding proteins by affinity proteomics

**Figure 4:**
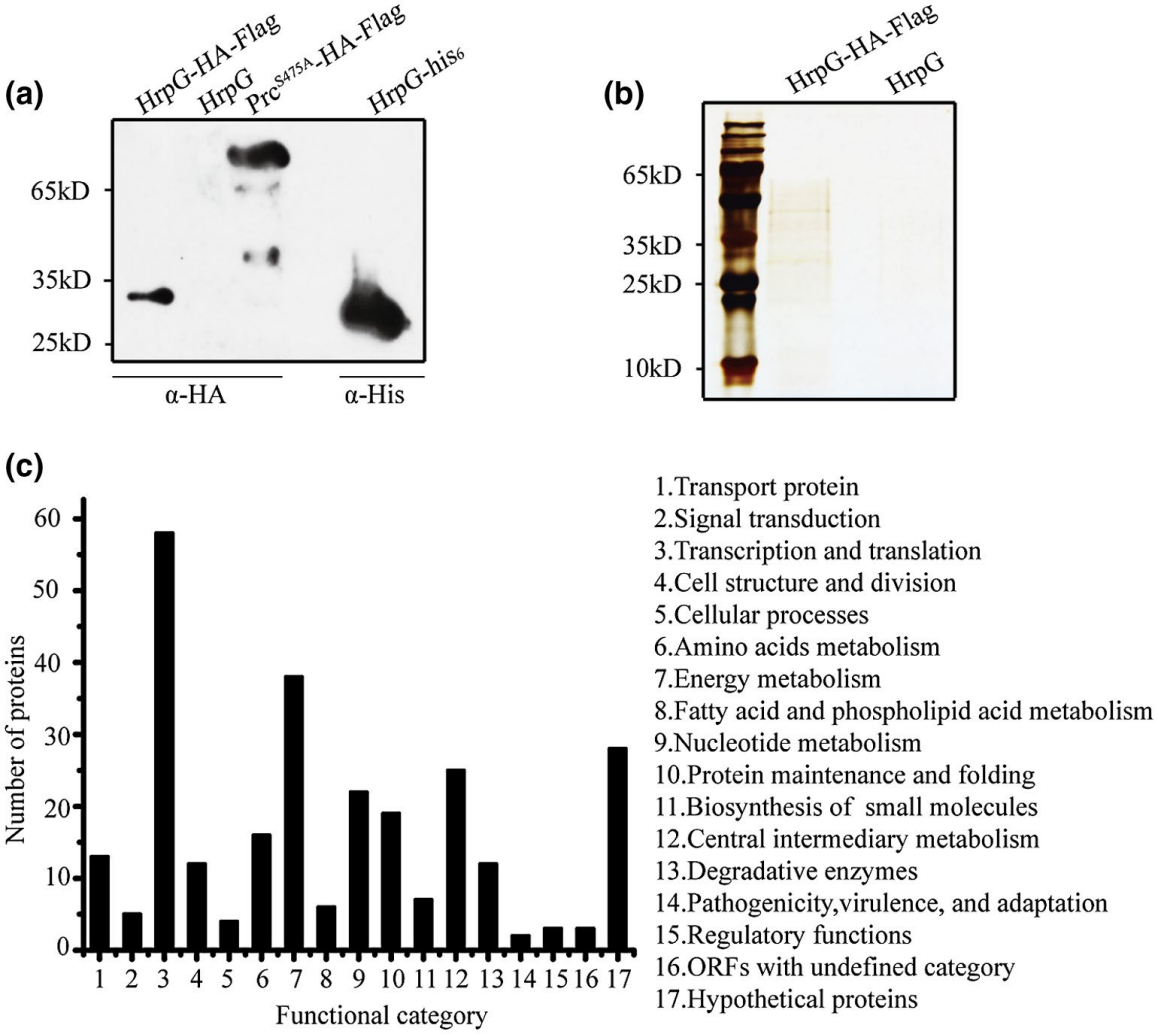
Tandem affinity purification (TAP) identifies HrpG‐interacting proteins. (a) Western blotting of the HrpG‐HA‐FLAG sample. Enriched TAP samples were detected using an anti‐haemagglutinin (HA) monoclonal antibody. The strain overexpressing *hrpG* by recombinant pHM1 vectors without any tag was used as a negative control. Point mutation of the gene encoding the Prc protease with the HA‐FLAG tag and full‐length HrpG with the C‐terminal His_6_ tag was used as a positive control. (b) TAP silver staining. The enriched TAP samples were separated by SDS‐polyacrylamide gel electrophoresis together with silver staining and analysed by nanoscale liquid chromatography‐mass spectrometry (nsLC‐MS/MS). (c) Functional categories of the putative HrpG‐binding proteins. Details of these proteins are given in Table S4

We employed an affinity proteomic approach using tandem affinity purification (TAP) together with nanoscale liquid chromatography‐mass spectrometry (nanoLC‐MS/MS) to screen potential HrpG‐binding proteins with the expectation that HrpG functions together with the other proteins. An Xcc strain in which the haemagglutinin (HA)‐FLAG tag was fused to the C‐terminal of HrpG and cloned into vector pHM1 in the ΔhrpG background (ΔhrpG‐hrpG‐HA‐FLAG) was constructed for the TAP analysis. The HA‐FLAG tags did not affect the bacterial phenotypes because the virulence level and the induction of the HR of the recombinant strain were similar to those of the ΔhrpG‐hrpG complementary strain (Figure S1a‐c). Western blotting confirmed the HrpG‐HA‐FLAG was expressed in vivo and that both HA and FLAG monoclonal antibodies were separately immunoprecipitated (Figure S1e,f). The procedures used to culture and collect the cells of the ΔhrpG‐hrpG‐HA‐FLAG strain were the same as those used for the ChIP assay. To prevent the dissociation of HrpG–protein interactions as much as possible, the collected Xcc strain was immediately lysed by freeze grinding. Through two independent rounds of immunoprecipitation, the eluted HrpG‐HA‐FLAG‐protein complex was subjected to western blotting (Figure 4a) to verify its availability before silver staining (Figure 4b). The parts of the sample gel that differed from the control were clipped and digested for nanoLC‐MS/MS analyses. The affinity proteomic screening detected 273 proteins that were classified into 17 functional categories (Figure 4c and Table S4). These proteins potentially bound to HrpG and their diversity indicated that HrpG might bind a variety of proteins to modulate bacterial T3SS expression and other important signalling pathways.

Besides the undefined and hypothetical proteins, the most abundant five GO functional categories were transcription and translation (58/273), energy metabolism (38/273), central intermediary metabolism (25/273), nucleotide metabolism (22/273), and amino acids metabolism (16/273), which indicated HrpG may participate widely in bacterial basal metabolism. Notably, the NADH‐ubiquinone oxidoreductase subunits NQO1–NQO6 that are the components of respiratory complex I (Yagi and Matsuno‐Yagi, 2003) were identified in the TAP data, indicating that HrpG was associated with the bacterial electron transfer chain and may help to provide the proton‐motive force required for ATP synthesis. Genes that encode the pilin (XC1058), flagellar (XC2245), and fimbrial (XC0941) assemblies were identified in the TAP data, suggesting that HrpG may participate in bacterial motility. This result is consistent with a previous report that the *hrpG* mutant showed higher swarming ability in a nutrient‐limited condition (Guo *et al.*, 2011). Furthermore, the gene that encodes c‐di‐GMP phosphodiesterase A (XC2324) was identified in the TAP data, suggesting that HrpG may take part in the degradation of the ubiquitous second messenger signalling molecule c‐di‐GMP (cyclic di‐GMP), and thus regulate bacterial virulence, cell cycle, and biofilm formation (Opoku‐Temeng and Sintim, 2017). Five two‐component signalling system proteins encoded by XC2229, XC3452, XC0850, XC4031, and XC3057 were identified in the TAP data and were different from nine genes identified in the ChIP‐seq data. This confirmed that, besides HrpG being involved in regulating the expression of genes in two‐component signalling systems, these systems also help to refine the functions of HrpG under certain specific living conditions.

### 2.4 Histone‐like protein HU_xcc_ binds to HrpG and is involved in bacterial virulence

**Figure 5:**
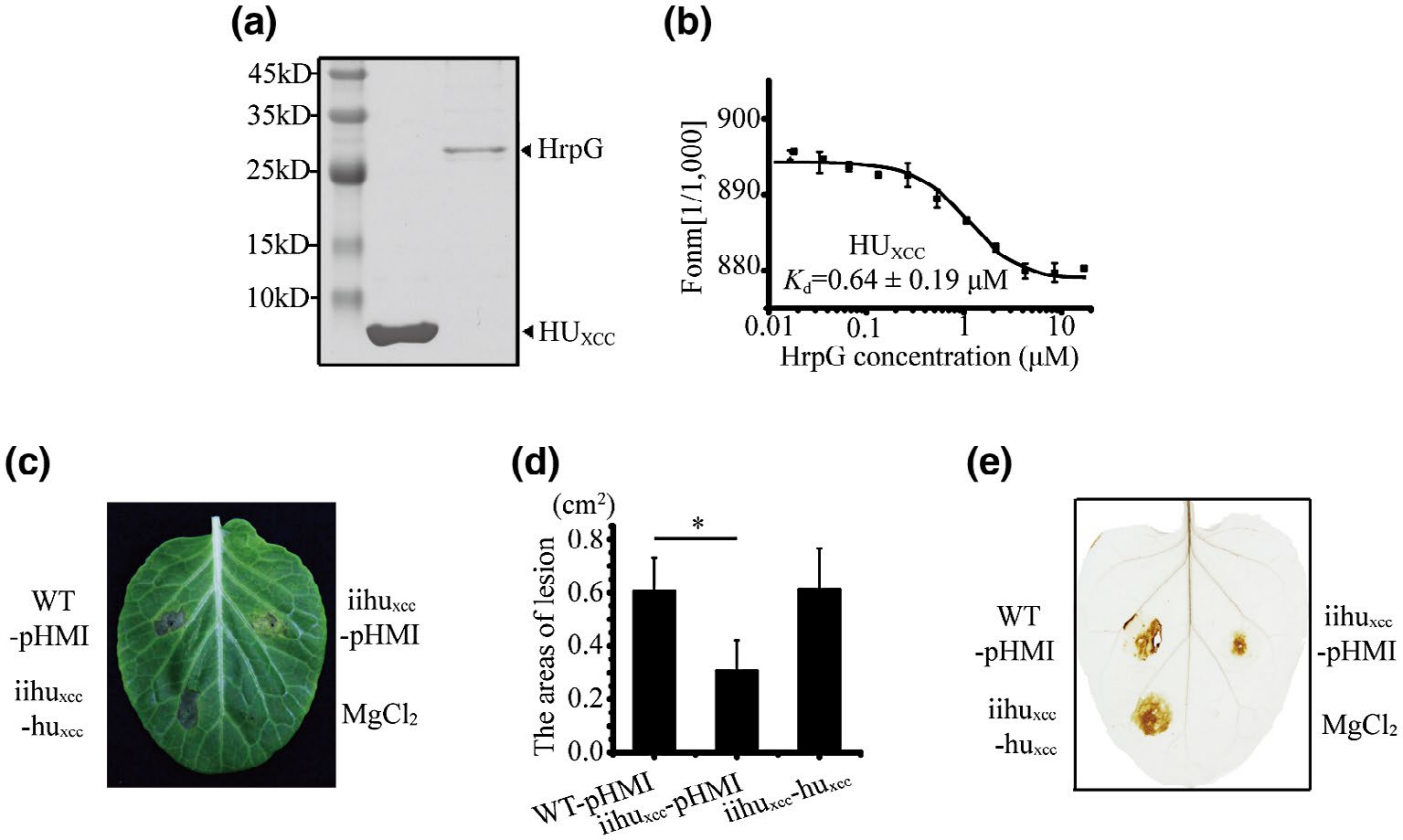
Histone‐like protein HU_xcc_ binds to HrpG and is involved in bacterial virulence. (a) Purification of the HrpG and HU_xcc_ proteins. The proteins were separated by SDS‐polyacrylamide gel electrophoresis (SDS‐PAGE) before staining with Coomassie brilliant blue. (b) Microscale thermophoresis analyses revealed that HU_xcc_ binds HrpG proteins. HU_xcc_ was labelled and the titrations of HrpG ranged from 0.001 to 17 μM. The solid curve is the fit of the data points to the standard KD‐Fit function. Black bars indicate standard deviations. *K*_d_ is the dissociation constant. The experiment was repeated three times. (c) Virulence assay of *Xanthomonas campestris* pv. *campestris* (Xcc) against host cabbage (*Brassica oleracea* ‘Jingfeng No. 1’). Insertion inactivation mutant of *hu_xcc_* (ΔHU_xcc_) partially weakened the virulence, whereas the wild type (WT) and complementary strains of *hu_xcc_* did not. Bacterial strains were inoculated onto cabbage, with 10 mM MgCl_2_ used as a negative control. (d) Virulence level of bacterial strains. The areas of lesion were recorded 5 days after inoculation. Vertical bars indicate the standard deviation (*n* = 5). Asterisks indicate significant differences compared with the WT (Student's *t* test, *p* ≤ .05). (e) Hypersensitive response (HR) assay of bacterial strains. The bacteria lost their ability to produce an HR in a nonhost plant (*Nicotiana tabacum* ‘SR1’) to some degree. Bacterial strains were inoculated onto nonhost tobacco, with 10 mM MgCl_2_ used as a negative control

The TAP analysis identified a DNA‐binding histone‐like protein HU (XC3262; HU_xcc_). The low molecular weight HU proteins (approximately 90 amino acids) are abundant DNA‐binding proteins with no binding specificity (Ptashne, 1986) and are critical for the maintenance of the nucleoid structure and function (Grove, 2011). HUs are involved in almost all DNA‐dependent functions, including repair, replication, recombination and transcription, and transposition in prokaryotes (Dorman and Deighan, 2003; Kamashev *et al.*, 2008). We heterogeneously expressed HU_xcc_ in *Escherichia coli* BL21(DE3) using a recombinant pET30a vector and purified the protein product by affinity chromatography with Ni‐NTA (nickel‐nitrilotriacetic acid) agarose beads (Figure 5a). In vitro MST assay experimentally verified that HU_xcc_ physically bound to HrpG with a dissociation constant (*K*_d_) of 0.64 ± 0.19 μM (Figure 5b), suggesting a high affinity level for the HU_xcc_–HrpG interaction.

We constructed insertional inactivation mutants of *hu_xcc_* (Δhu_xcc_) by pK18mob suicide vector integration, and characterized the phenotype of the Δhu_xcc_ mutants, which exhibited significantly decreased virulence levels compared with the WT Xcc (Figure 5c,d). The areas of lesion after infection with Δhu_xcc_ were 0.3 ± 0.11 cm^2^, which is significantly less than that of the WT (0.6 ± 0.13 cm^2^, *p* < .05). Genetic complementation (Δhu_xcc_‐hu_xcc_) restored the virulence deficiency (0.6 ± 0.15 cm^2^). The HR symptom induced by Δhu_xcc_ also was remarkably alleviated compared with the WT and complementary strains (Figure 5e). These results indicate that *hu_xcc_* is involved in bacterial virulence in the host cabbage and HR.

## 3 DISCUSSION

In the plant pathogenic bacteria *Xanthomonas* spp., HrpG, which tops the T3SS regulatory hierarchy (Wengelnik *et al.*, 1996), has a dominant role in bacterial virulence and its expression and regulation have been studied for years (Ficarra *et al.*, 2015; Zhang *et al.*, 2018). In the present study, we used ChIP‐seq (Figure 1) and TAP (Figure 4) to detect genes that were directly regulated by HrpG and proteins that potentially interacted with HrpG. A consensus HrpG‐binding motif was confirmed for the first time (Figure 1b). The identification and confirmation of *hpa2*, *hrcU*, and *hrpE*, which have been reported to be *hrp* genes controlling bacterial virulence (Zou *et al.*, 2006), confirmed the reliability of our ChIP‐seq data. Consistent with previous studies (Li *et al.*, 2011), we found that *hpa2* was positively regulated by HrpG. Although it is widely acknowledged that HrpG indirectly regulates *hrpB‐hrpF* operons in the *hrp* genes regulatory cascade in which HrpG regulates *hrpX* and *hrpA*, and then HrpX regulates the other *hrp* genes in *hrpB‐hrpF* operons (Frederick *et al.*, 2001; Huang *et al.*, 2009), our results showed that HrpG also directly and positively regulated the transcript level of *hrcU* (XC3012, a representative of the *hrpC* operon) and *hrpE* (XC3021, a representative of the *hrpE* operon). These results suggest that *hrpB‐hrpF* operons can be regulated directly by HrpG, as well as by HrpX, and indicate a novel mechanism for differential regulation of HrpG and HrpX.

An extensive comparison of our ChIP‐seq data with genome‐wide microarray data (Guo *et al.*, 2011) provided some insights to define the HrpG regulon. Although the two data sets are for Xcc and Xac, respectively, they are comparable because of the high similarity between the Xcc and Xac genomes in which >80% of genes are conserved between Xcc and Xac (Da Silva *et al.*, 2002). We detected 186 candidate HrpG downstream genes in our ChIP‐seq analysis and 232 differential expressed genes in the microarray analysis. This difference in gene numbers can be explained by noting that the microarray data contained genes that were indirectly as well as directly regulated by HrpG, whereas the ChIP‐seq data contained only genes with promoter regions that were bound directly by HrpG (i.e. direct regulation). The 186 HrpG downstream genes in the ChIP‐seq data were assigned to 15 categories and annotated with GO terms and KEGG pathways (Figure 1e), and the 232 genes in the microarray data were assigned to 18 functional categories and annotated with the J. Craig Venter Institute (JCVI) role categories. Among them, 13 categories overlapped: amino acid biosynthesis; biosynthesis of cofactors, prosthetic groups, and carriers; cell structure and division; cellular processes; central intermediary metabolism; DNA metabolism; energy metabolism; fatty acid and phospholipid acid metabolism; mobile genetic elements; regulatory functions (separated as signal transduction and regulatory functions for genes in the ChIP‐seq data); transcription; transport protein; and hypothetical proteins (i.e. unclassified proteins, unknown function proteins, hypothetical proteins and not in JCVI in microarray data). The category protein fate was not assigned to genes in the ChIP‐seq data and the category pathogenicity, virulence, and adaptation was not assigned to genes in the microarray data. Only nine genes were common in both the ChIP‐seq and microarray databases: endopolygalacturonase (XAC0601, XC0705), type III secretion system protein HrcU (XAC0406, XC3012), HrcS protein (XAC0401, XC3017), phosphate regulon transcriptional regulator phoU (XAC1573, XC2712), response regulator (XAC2897, XC1383), ABC transporter permease, nrtB (XAC0827, XC3459), Hpa2 protein (XAC0417, XC3001), and HrpE protein (XAC0397, XC3021). Notably, 17.2% (32 of 186) and 53.4% (124 of 232) of the genes of the HrpG regulon were classified as hypothetical or unknown function proteins in the ChIP‐seq and microarray data sets, respectively. This probably reflects the complete annotation of the Xcc strain 8004 genome (Qian *et al*., 2005), which will improve as more genetic and biochemical data become available. The whole *hrp* gene cluster of 24 genes was detected in the microarray data, including *hrp*, *hrc*, and *hpa*, and most known T3SS effector genes were identified and found to be down‐regulated in *hrpG* mutants. Conversely, only three T3SS genes, *hrpE*, *hrcU*, and *hpa2*, were identified in the ChIP‐seq data. These results indicate that most of the T3SS genes were indirectly controlled by HrpG, which is consistent with what is known about the HrpG‐HrpE downstream genes regulatory cascade. Nineteen genes encoding T2SS substrates and eight genes involved in flagellar biosynthesis were identified only in the microarray data and not in the ChIP‐seq data, indicating that HrpG controls the T2SS and flagellar biosynthesis without binding directly to the promoters of these genes.

The yeast two‐hybrid (Y2H) system has been used to identify HrpG‐interacting proteins in Xac (Alegria *et al.*, 2004). However, for bacterial research, Y2H is an in vitro method that produces a high rate of false positives and only high‐affinity interactions can be retained (Mehla *et al.*, 2018). Co‐immunoprecipitation (Co‐IP) is a traditional and powerful technique that has been used to identify native and physiological protein–protein interactions (Free *et al.*, 2009). However, Co‐IP has several obvious disadvantages: a suitable and specific antibody must be available, transient interactions are difficult to capture, and the acquired interactions may be indirect, so verification is tedious (Ngounou Wetie *et al*., 2014). In a previous study, we used the TAP approach to identify the substrates of Prc protease in *X. campestris* (Deng *et al.*, 2018). In the present study, we used TAP for the HrpG interactome screening. The HrpG protein fused with two different tags in tandem was expressed in Xcc and purified with its interaction partners under native conditions, thereby maintaining the complex integrity. Two rounds of affinity chromatography purification were used to guarantee high specificity and low numbers of false positives (Oeffinger, 2012). We identified 273 putative HrpG‐interacting proteins from the TAP data (Figure 4). One intriguing result of the data analysis was that the putative sensor histidine kinase (XC3670) of HrpG (Li *et al.*, 2014) was absent in the TAP database. One possible explanation is that the hybrid kinase–response regulator interaction was weak and transient, making it was hard to capture and fix the potential interaction throughout the pipeline. The TAP experimental strain with conserved phosphorylation sites (ΔhrpG‐hrpG^D61A^‐HA‐FLAG) substituted would be better suited for fixing the transfer process of the phosphoryl group.

The bacterial histone‐like HU proteins are preferred to chromosomal DNA containing structural aberrations and bind to double‐stranded (ds) DNA without sequence specificity (Dorman and Deighan, 2003; Swinger and Rice, 2004). HUs can introduce structural changes to the DNA because of their ability to bend, loop, and compact DNA (Drlica and Rouviere‐Yaniv, 1987); however, the roles of HUs in the bacterial T3SS have been less investigated and reported. We identified a DNA‐binding histone‐like HU protein (XC3262, HU_xcc_) in the TAP data that interacts with the T3SS master regulator HrpG in vivo and in vitro (Figure 5b) and is involved in T3SS‐associated bacterial virulence (Figure 5c‐e). We speculate that HU_xcc_ possibly introduces structural changes in the promoter regions of genomic DNA and acts as a co‐activator of HrpG, then facilitates HrpG activation of downstream gene expression upon induction. Although further genetic and biochemical experiments are needed to precisely determine the contribution of interacting partners to HrpG regulation, the results of this study revealed the genome‐wide HrpG regulon that is directly bound by HrpG, the consensus HrpG‐binding motif, and in vivo HrpG interacting proteins that are involved in HrpG function. These data will contribute to understanding the bacterial signalling pathways in HrpG‐regulated T3SS.

## 4 EXPERIMENTAL PROCEDURES

### 4.1 Bacterial strains and culture conditions

The bacterial strains and plasmids used in this study are listed in Table S1. Xcc strains were routinely grown at 28 °C in rich medium NYG (5 g/L tryptone, 3 g/L yeast extract, and 20 g/L glycerol, pH 7), minimal and induction medium XVM2 (5 g/L glucose, 1 g/L sodium citrate, 2 g/L (NH_4_)_2_SO_4_, 4 g/L K_2_HPO_4_, 6 g/L KH_2_PO_4_, and 0.2 g/L MgSO_4_, pH 7.0) and XCM2 medium (2.36 g/L succinic acid, 0.15 g/L casamino acids, 1 g/L (NH_4_)_2_SO_4_, 0.001 g/L MgSO_4_, 10.5 g/L K_2_HPO_4_, and 8.35 g/L KH_2_PO_4_, pH 6.6). *E. coli* DH5α, cultured at 37 °C in Luria–Bertani (LB) medium (10 g/L tryptone, 5 g/L yeast extract, 10 g/L NaCl, pH 7), was used to prepare all recombinant vectors needed. *E. coli* BL21(DE3) and *E. coli* M15 were used for protein expression. When required, the following concentrations of antibiotics were used: 100 μg/ml ampicillin, 50 μg/ml kanamycin, 100 μg/ml spectinomycin, and 25 μg/ml rifampicin. Electroporation was performed in a Bio‐Rad Pulser XCell at 18 kV/cm, 25 μF, and 200 Ω. All other general molecular biology operations were carried out according to standard molecular cloning protocols.

### 4.2 Bacterial genetic manipulation

In‐frame deletion mutants were constructed using the suicide vector pK18mobsacB by homologous double‐crossover recombination according to previous studies (Burckstummer *et al.*, 2006; Wang *et al.*, 2016). The insertional inactivation mutants used the suicide vectors pK18mob by homologous single‐crossover methodology (Qian *et al.*, 2008; Kang *et al.*, 2015). His_6_‐tagged proteins were expressed using pET30a (Novagen) vectors and pQE30Xa (Qiagen) according to the manufacturer's instructions. To construct genetic complementation strains, the broad‐host vector pHM1 with inserts of full‐length sequences of genes of interest (under the control of the *lacZ* promoter) were established and electroporated into Xcc competent cells. The primers used to amplify the sequences are listed in Table S2.

### 4.3 Hypersensitive response and virulence assay

HR assays were carried out by inoculating bacterial cultures onto leaves of 6‐week‐old tobacco plants (*N. tabacum* ‘SR1’) using sterile injectors. The bacterial strains, cultured in NYG medium at 28 °C until OD_600_ = 0.8 ± 0.01, were washed twice with 10 mM MgCl_2_ before being inoculated. The infected leaves were harvested and carefully transferred to culture dishes to conduct the 3,3‐diaminobenzidine (DAB) staining experiment 36 hr after inoculation. The DAB solution (0.005% Tween 20 (vol/vol), 3.58 g/L Na_2_HPO_4_, and 1 g/L DAB) was added until the leaves were completely covered and the samples were incubated in the dark for at least 5 hr at room temperature with mild agitation. Finally, the destaining solution was poured into the culture dishes and the leaves were dipped for at least 24 hr to clear the chlorophyll after discarding the DAB solution.

Bacterial strains were cultured in NYG medium containing appropriate antibiotics at 28 °C until OD_600_ = 0.4 ± 0.01. The virulence assay was performed by inoculating 1 ml bacterial solution onto leaves of 8‐week‐old cabbage plants (*B. oleracea* ‘Jingfeng No. 1’) using sterile injectors. Before inoculation, the bacterial strains were washed with 10 mM MgCl_2_ and diluted 10 times. At 5 days after inoculation, the virulence level of bacterial strains was estimated by measuring the lesion area of leaves.

### 4.4 Protein expression, purification, and western blotting

HrpG‐His_6_ recombinant proteins were expressed by constructing the corresponding recombinant pQE30Xa (Qiagen) expression vectors and transforming them into *E. coli* M15 cells. The primers used to generate these constructs are listed in Table S2. For protein expression, overnight cultures of each strain were inoculated into 1 L LB medium and grown until they reached OD_600_ = 0.5, and induced with 0.8 mM isopropyl‐β‐D‐thiogalactopyranoside (IPTG) for 4 hr at 20 °C. The bacterial cultures were collected, sonicated, centrifuged, and used in Fast Protein Liquid Chromatography AKTA Purifier 10 with Frac‐900 (GE Healthcare). The ATKA system was pre‐equilibrated with 300 mM NaCl and 50 mM sodium phosphate buffer, pH 7 at a flow rate of 1 ml/min and then applied to a HisTrap HP column to separate target impure protein. The elution profiles were collected by absorbance at 280 nm and confirmed by SDS‐polyacrylamide gel electrophoresis (SDS‐PAGE) and western blotting. Purified proteins were concentrated using Centricon YM‐10 columns (Millipore) and the elute buffer was changed into storage buffer for further use (50 mM Tris‐HCl, 0.5 mM EDTA, 50 mM NaCl, 5% glycerol).

A prokaryotic expression system with vector pET30a and *E. coli* BL21 (DE3) (Novagen) was used to express recombinant HU_Xcc_‐His_6_. Protein was expressed and purified by affinity chromatography using Ni‐NTA agarose beads (Novagen) according to the manufacturer's manual. In brief, the expression strains were inoculated in rich Luria Bertani medium at 37 °C, grown to OD_600_ = 0.4–0.8, and induced at 16 °C for 16 hr with 1 mM IPTG.

Western blotting was performed by transferring the proteins onto polyvinylidene fluoride (PVDF) membrane (Millipore). Monoclonal antibodies of 3 × FLAG or HA (M20003‐L) and His_6_ tags (M20001L, Abmart, China) were used to detect the proteins. All of the antibodies were diluted 5,000–10,000‐fold before use.

### 4.5 Electrophoretic mobility shift assay

To detect HrpG‐DNA binding, DNA duplex fragments corresponding to the sequences upstream of targeted genes were PCR‐amplified and purified. They were labelled with [γ‐^32^P]ATP using T4 polynucleotide kinase (Fermentas) and purified with a ProbeQuant G‐50 column (GE Healthcare) that removed free [γ‐^32^P]ATP. The binding assays were carried out in 20 μl volumes of reaction buffer (10 mM Tris–HCl, pH 7.5, 50 mM KCl, 1 mM dithiothreitol and 1 μl of 50 ng/μl poly(dI:dC)). DNA probe (5 fmol) and purified protein (0–40 μM) were incubated together for 30 min at 28 °C. For the competition reaction, a certain amount of unlabelled DNA probe was co‐incubated for 30 min at room temperature before electrophoresis. To stop the reaction, 3.3 μl 6 × DNA loading buffer was added. Finally, the samples were loaded onto a 5% native PAGE gel and the electrophoresis was performed under 120 V for 50 min with 0.5 × TBE buffer (5.4 g/L Tris, 2.75 g/L boric acid and 2 ml/L 0.5 M EDTA, pH 8) before autoradiography.

### 4.6 Microscale thermophoresis assays

The MST measurement was used for detecting protein–protein interactions. In brief, 10 μM purified protein HU_Xcc_ was labelled with a Monolith NT Protein Labeling Kit RED‐NHS (Nano Temper Technologies GmbH) using red fluorescent dye NT‐647 N‐hydroxysuccinimide (amine‐reactive) according to the manufacturer's instructions. The additional labelling reagents were removed by buffer‐exchange column chromatography and the labelled protein HU_Xcc_ was eluted in NTA buffer (300 mM NaCl and 50 mM sodium phosphate buffer, pH 7). The binding reactions were carried out on a Monolith NT.115 Microscale Thermophoresis instrument (Nano Temper Technologies GmbH) using standard treated capillaries. Equal quantities of labelled protein were titrated by the purified HrpG and exchanged into the 1 × MST buffer (50 mM Tris, 150 mM NaCl, 10 mM MgCl_2_, pH 6) with 0.05% Tween using a 1:1 series dilution method. The KD‐Fit function of the Nano Temper Analysis software v. 1.5.41 was used to fit the curve and determine the value of the dissociation constant (*K*_d_).

MST was also used to quantify the binding affinities between protein and DNA. 5′‐FAM‐labelled oligonucleotide primers were synthesized by the Beijing Ruibiotech Company (China) and were annealed with an unlabelled complementary primer to form dsDNA. The labelled dsDNA was added to serially diluted protein reaction volumes containing 50 mM Tris–HCl (pH 7.4), 150 mM NaCl, 10 mM MgCl_2_, and 0.05% (vol/vol) Tween‐20. The curves were fitted by Nano Temper Analysis v. 1.5.41 from three replicates, and the value of the dissociation constant (*K*_d_) was calculated.

### 4.7 RNA extraction and RT‐qPCR analysis

The concentration of total RNA, isolated from bacteria using TRIzol (Invitrogen), was determined using a NanoDrop 1000 spectrophotometer (Thermo Fisher). Any DNA contamination was removed by digestion with RNase‐free DNase I (Ambion). First‐strand cDNA was synthesized using random primers (Promega) and Superscript III reverse transcriptase (Invitrogen). The negative control lacked reverse transcriptase to determine if there was any contaminating DNA, while the positive control included DNA from the WT bacterial strain. mRNA levels of target genes were quantified by RT‐qPCR using Maxima SYBR Green (Fermentas) and the CFX96 Real‐time PCR Detection System (Bio‐Rad). The cDNA prepared from tRNA served as the internal control and the reference. The RT‐qPCR primers are listed in Table S2.

### 4.8 ChIP‐seq

The protocol used for ChIP followed that of a previous study (Pan *et al.*, 2017). Briefly, bacterial strains were grown in NYG medium until OD_600_ = 0.4 ± 0.05. After 2 hr of induction with equal volume XCM2 medium, the cells were collected. The samples were cross‐linked with 1% formaldehyde and subsequently quenched with 0.5 M glycine for 10 min. Bacterial cultures (1 L), harvested by centrifugation, were washed with cold phosphate‐buffered saline (PBS; 0.27 g/L KH_2_PO_4_, 1.42 g/L Na_2_HPO_4_, 8 g/L NaCl, and 0.2 g/L KCl, pH 7.4) twice, and then resuspended in lysis buffer (10 mM Tris, pH 8.0, 20% sucrose, 50 mM NaCl, 10 mM EDTA, pH 8.0, 10 mg/ml lysozyme, and 1 mM PMSF). Immunoprecipitation (IP) buffer (50 mM HEPES‐KOH, pH 7.5, 150 mM NaCl, 1 mM EDTA, pH 8.0, 1% Triton X‐100, 0.1% sodium deoxycholate, and 0.1% SDS) was added to the bacterial cell suspension, and the cells sonicated using a Bioruptor (Diagenode) to generate DNA fragments (150–300 bp). The cell lysis was precleared with 20 μl protein A sepharose (GE) for 30 min at 4 °C on a rotator, and 400 μl aliquots were retained as the loading control DNA (input sample). For the ChIP assays, 50 μl protein A sepharose (50% slurry) and 2 μl anti‐His_6_ monoclonal antibody (Abmart) was added to a 1 ml aliquot of the DNA sample. The mixture was slowly rotated at 4 °C overnight. The protein A sepharose beads were collected and washed twice with immunoprecipitation buffer, and subsequently once each with wash buffer (10 mM Tris–HCl, pH 8, 250 mM LiCl, 1 mM EDTA, pH 8, 0.5% Nonidet‐P40, and 0.5% sodium deoxycholate), high salt wash buffer (50 mM HEPES, pH 7.9, 500 mM NaCl, 1 mM EDTA, pH 8, 0.1% SDS, 1% Triton X‐100, and 0.1% deoxycholate) and TE buffer (10 mM Tris‐HCl, pH 8, and 1 mM EDTA). The immunoprecipitated chromatin was removed from the beads by adding 100 μL of elution buffer (50 mM Tris‐HCl, pH 7.5, 10 mM EDTA, and 1% SDS), and the solution was incubated for 10 min at 65 °C. RNase A and proteinase K were used to remove RNA and protein, respectively. Furthermore, the DNA was extracted with 24:1 (vol/vol) chloroform:isoamyl alcohol, and precipitated with ethyl alcohol. Finally, the DNA was purified using a PCR purification kit (Qiagen) after using a 2% agarose gel. High‐throughput sequencing was performed using the Illumina Highseq‐2000 system by the Beijing Institute of Genomics genomic service. The resulting high‐throughput sequencing reads were analyzed by the Burrows–Wheeler aligner method. The cleaned reads were aligned to the genomic sequence database of Xcc 8,004. Peak calling was conducted by MACS257. The consensus HrpG‐binding motif analysis was completed using MEME and FIMI tools in the MEME software suite (Bailey *et al.*, 2015).

### 4.9 Tandem Affinity Purification

The TAP procedure was conducted following the protocol of the FLAG HA Tandem Affinity Purification Kit (Sigma‐Aldrich) and previous study (Deng *et al.*, 2018). In brief, the bacterial cells were collected and ground before being resuspended in lysis buffer (50 mM Tris‐HCl, pH 8.0, 0.15 M NaCl, 1 mM EDTA, 1 mM PMSF, and 1 tablet/20 ml protease inhibitor cocktail tablets [Roche]). Subsequently, they were centrifuged at 13,000 × g for 20 min at 4 °C, and the supernatant represented the whole cell lysate. Prewashed ANTI‐FLAG M2 resin was added to the sample lysate and the mixture was incubated from 2 hr to overnight at 4 °C on a rotator. The resin–protein complex was washed with lysis buffer using three rounds of low‐speed centrifugation. For each wash, the resin was lightly agitated in the lysis buffer, centrifuged at 3,000 × g for 1 min, and then the final wash volume was discarded. The remaining resin was transferred into a spin column, and 2.5 volumes of 3 × FLAG peptide (150 ng/μl) were added and co‐incubated for at least 10 min at 4 °C. The column was spun before eliminating the tip to a clean microcentrifuge tube and keeping the eluate, which contained the eluted protein. The elution process was repeated twice. Then, 40 μl prewashed ANTIHA resin slurry in lysis buffer was used and incubated for 30 min to 2 hr at 4 °C with gentle rocking. The supernatant was discarded after the incubation, and the ANTI‐HA resin–protein complex was washed with lysis buffer to remove unbound protein. Next, 50 μl of 8 M urea was added and co‐incubated for a minimum of 10 min at room temperature. The sample was spun at 3,000 × g for 1 min, and the eluate was prepared to load and separate on a 12% SDS‐PAGE gel. The differential gel bands between sample and control were manually excised after silver staining, and each band then underwent enzymatic digestion and subsequent LC‐MS/MS identification.

The nanoLC‐MS/MS identification of proteins was performed on a Thermo Finnigan LTQ linear ion trap mass spectrometer in accordance with a Thermo Finnigan Surveyor MS Pump Plus HPLC system. Tryptic peptides were loaded onto a trap column (300 SB‐C18, 5 × 0.3 mm, 5‐μm particle; Agilent Technologies). The peptides were eluted over gradient solution C (80% acetonitrile and 0.1% formic acid) at a flow rate of 500 nl/min and introduced into the online linear ion trap mass spectrometer (Thermo Fisher) using nano‐electrospray ionization. The five most abundant ions (one microscan per spectra) were selected for fragmentation from a full‐scan mass spectrum by collision‐induced dissociation for data‐dependent scanning.

MS data were analysed with SEQUEST against Xcc 8004 protein database in NCBI and displayed with Bioworks v. 3.2. Peptides with +1, +2, or +3 charge states and with cross correlations of ≥1.90, >2.5, and >3.0, respectively, were accepted. Carbamidomethylation on cysteine and oxidation on methionine were selected as residue modifications. SEQUEST was searched with a peptide tolerance of 3.0 AMU and a fragment of 1.0 AMU.

## Supporting information


**Fig. S1** The His_6_ and HA‐Flag tag did not influence bacterial virulence. (A) Virulence assay of *Xanthomonas campestris pv. campestris (Xcc)* against host cabbage (*Brassica oleracea* cv. Jingfeng No. 1). Bacterial strains were inoculated onto host cabbage, with 10 mM MgCl_2_ used as a negative control. (B) Virulence levels of bacterial strains was measured. The areas of lesion were recorded 5 days after inoculation. Vertical bars represent the standard deviation (*n* = 5). Asterisks indicate significant difference compared with with‐type (WT) *Xcc* (Student's t‐test, *p* ≤ 0.05). (C) Hypersensitive response assay of bacterial strains. The labeled and WT *Xcc* produced a hypersensitive response in the non‐host plant (*Nicotiana tabacum* cv. SR1). Bacterial strains were inoculated onto non‐host tobacco, with 10 mM MgCl_2_ used as a negative control. (D)–(F) Validation of tags used in the study by western blotting. The *hrpG* with His_6_ and HA‐Flag tags were provided *in trans* using recombinant pHM1 vectors.

**Fig. S2** Verification of the *hrpG*‐binding promoters. (A) Biotin‐labelled EMSA screenings. HrpG bound directly to the promoter regions of HrpG downstream genes. (B) Isotopic‐labelled EMSA screenings. EMSA was used to determine the binding events between HrpG and DNA probes labeled with [γ‐^32^P] ATP. The PCR products of the promoter regions of genes XC0124, XC0125, XC1298, XC1050, XC1164 and XC3076 were used as probes. Each assay was repeated three times.

**Table S1** Bacterial strains and plasmids used in this study.

**Table S2** Primers used in this study.

**Table S3** ChIP‐seq analysis revealed HrpG regulated genes of *Xanthomonas campestris* pv. *campestris* grown in the XCM2 inducing medium.

**Table S4** Proteins identified by tandem affinity purification (TAP) under XCM2‐induced condition.

## Data Availability

The data that support the findings of this study are available from the corresponding author upon reasonable request.
