# Supplementary figures and images for: Analysis of HrpG regulons and HrpG‐interacting proteins by ChIP‐seq and affinity proteomics in Xanthomonas campestris
Source: Mol Plant Pathol. 2020 Jan 8;21(3):388–400. doi: 10.1111/mpp.12903 (PMC7036363; doi:10.1111/mpp.12903)

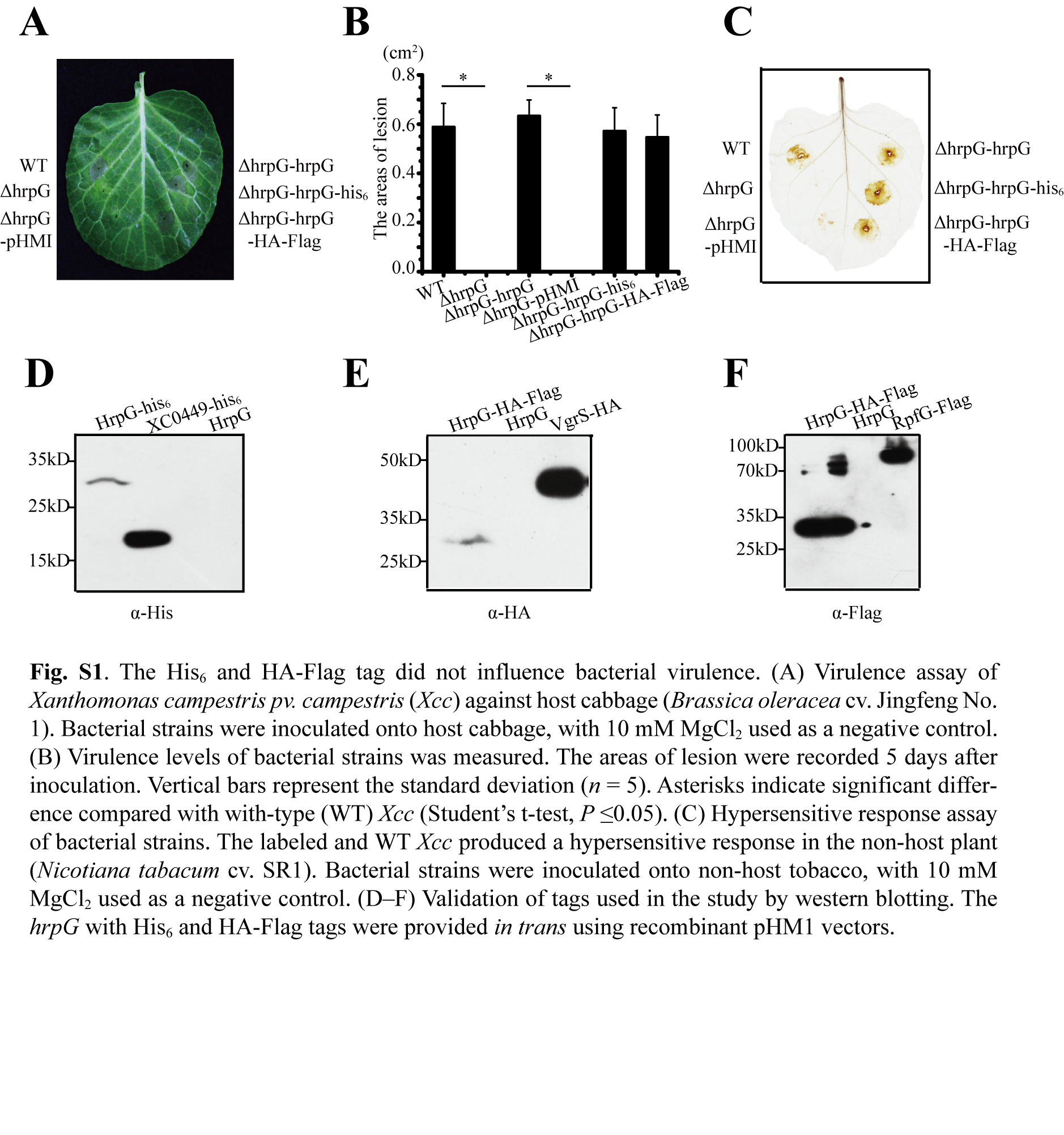

Supplement: Supplementary file 1 — Fig. S1 The His6 and HA‐Flag tag did not influence bacterial virulence. (A) Virulence assay of Xanthomonas campestris pv. campestris (Xcc) against host cabbage (Brassica oleracea cv. Jingfeng No. 1). Bacterial strains were inoculated onto host cabbage, with 10 mM MgCl2 used as a negative control. (B) Virulence levels of bacterial strains was measured. The areas of lesion were recorded 5 days after inoculation. Vertical bars represent the standard deviation (n = 5). Asterisks indicate significant difference compared with with‐type (WT) Xcc (Student's t‐test, p ≤ 0.05). (C) Hypersensitive response assay of bacterial strains. The labeled and WT Xcc produced a hypersensitive response in the non‐host plant (Nicotiana tabacum cv. SR1). Bacterial strains were inoculated onto non‐host tobacco, with 10 mM MgCl2 used as a negative control. (D)–(F) Validation of tags used in the study by western blotting. The hrpG with His6 and HA‐Flag tags were provided in trans using recombinant pHM1 vectors. [file MPP-21-388-s001.tif]

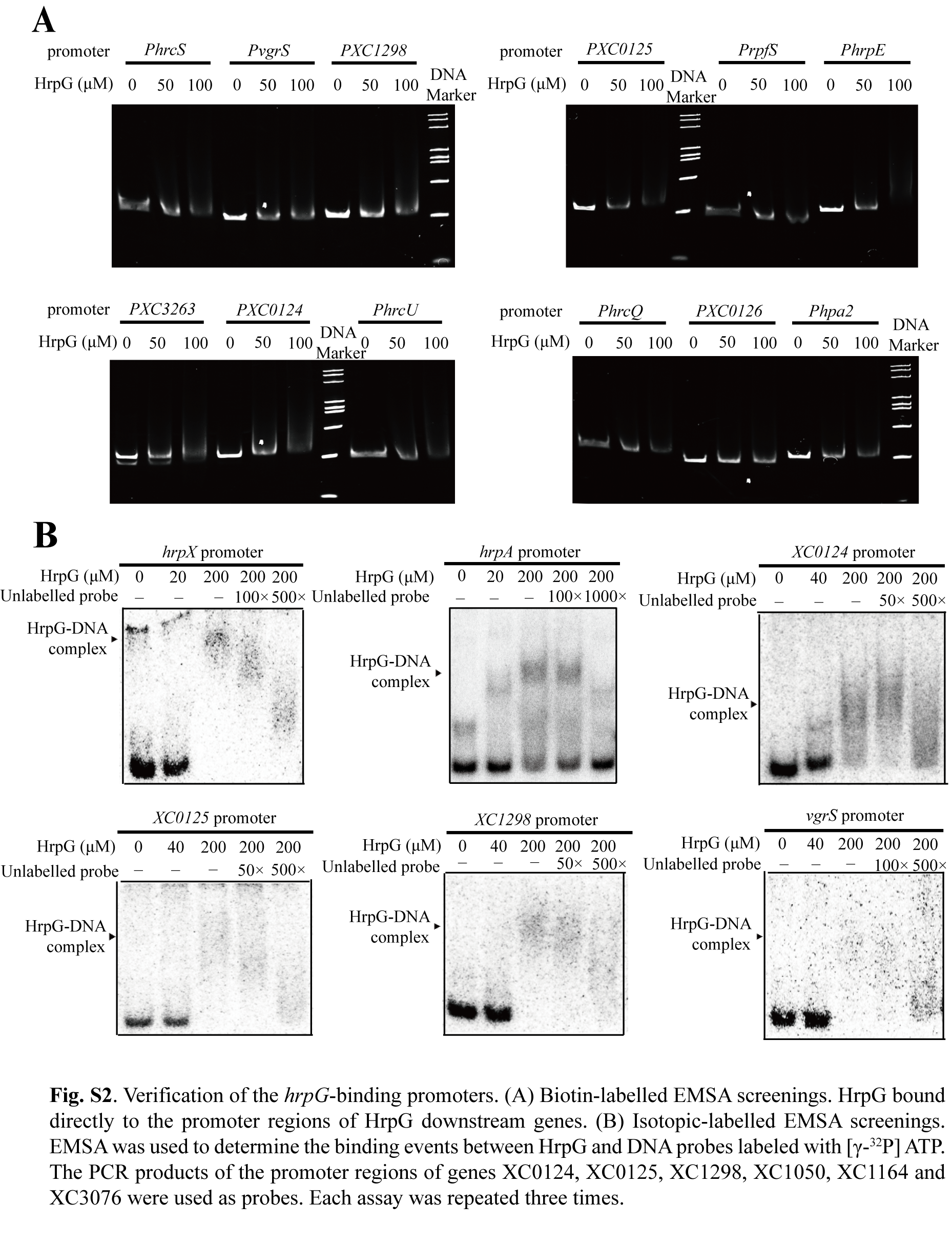

Supplement: Supplementary file 2 — Fig. S2 Verification of the hrpG‐binding promoters. (A) Biotin‐labelled EMSA screenings. HrpG bound directly to the promoter regions of HrpG downstream genes. (B) Isotopic‐labelled EMSA screenings. EMSA was used to determine the binding events between HrpG and DNA probes labeled with [γ‐32P] ATP. The PCR products of the promoter regions of genes XC0124, XC0125, XC1298, XC1050, XC1164 and XC3076 were used as probes. Each assay was repeated three times. [file MPP-21-388-s002.tif]
