# Supplementary material for: Analysis of HrpG regulons and HrpG‐interacting proteins by ChIP‐seq and affinity proteomics in Xanthomonas campestris
Source: Mol Plant Pathol. 2020 Jan 8;21(3):388–400. doi: 10.1111/mpp.12903 (PMC7036363; doi:10.1111/mpp.12903)
Supplement: Supplementary file 3 — Table S1 Bacterial strains and plasmids used in this study. [file MPP-21-388-s003.docx]

**Table S1.** Bacterial strains and plasmids used in this study.

| Strain or plasmid | Genotype or description | Resource or reference |
| --- | --- | --- |
| *E. coli* strains |  |  |
| DH5α | Host strain used for molecular cloning | Lab collection |
| BL21(DE3) | Host strain used for protein expression | Lab collection |
| M15 | Host strain used for protein expression | Lab collection |
| *Xanthomonas campestris* pv. *campestris* strains |  |  |
| 8004 | Wild type strain (WT), Rif^r^ | Lab collection |
| WT-pHM1 | WT containing a blank pHM1 vector, Rif^r^, Sp^r^ | This study |
| ΔhrpG | XC3077 (*hrpG*) in-frame deletion mutant, Rif^r^ | This study |
| Δhpa2 | XC3001 (*hpa2*) in-frame deletion mutant, Rif^r^ | This study |
| ΔhrcU | XC3012 (*hrcU*) in-frame deletion mutant, Rif^r^ | This study |
| ΔhrpE | XC3021 (*hrpE*) in-frame deletion mutant, Rif^r^ | This study |
| Δhu_xcc_ | XC3262 insertional mutant, Kan^r^ | This study |
| ΔhrpG-pHM1 | ΔhrpG containing a blank pHM1 vector, Rif^r^_,_ Sp^r^ | This study |
| Δhpa2-pHM1 | XC3001 (*hpa2*) in-frame deletion mutant containing a blank pHM1 vector, Rif^r^, Sp^r^ | This study |
| ΔhrcU-pHM1 | XC3012 (*hrcU*) in-frame deletion mutant containing a blank pHM1 vector, Rif^r^, Sp^r^ | This study |
| ΔhrpE-pHM1 | XC3021 (*hrpE*) in-frame deletion mutant containing a blank pHM1 vector, Rif^r^, Sp^r^ | This study |
| Δhu_xcc_ - pHM1 | iihu_xcc_ containing a blank pHM1 vector, Rif^r^_,_ Sp^r^ | This study |
| ΔhrpG-hrpG | Δ*hrpG* containing a pHM1::hrpG vector, Rif^r^, Sp^r^ | This study |
| ΔhrpG-hpa2 | Δ*hrpG* containing a pHM1::hpa2 vector, Rif^r^, Sp^r^ | This study |
| ΔhrpG-hrcU | Δ*hrpG* containing a pHM1::hrcU vector, Rif^r^, Sp^r^ | This study |
| ΔhrpG-hrpE | Δ*hrpG* containing a pHM1::hrpE vector, Rif^r^, Sp^r^ | This study |
| Δhpa2-hpa2 | Δ*hpa2* containing a pHM1::hpa2 vector, Rif^r^, Sp^r^ | This study |
| ΔhrcU-hrcU | Δ*hrcU* containing a pHM1::hrcU vector, Rif^r^, Sp^r^ | This study |
| ΔhrpE-hrpE | Δ*hrpE* containing a pHM1::hrpE vector, Rif^r^, Sp^r^ | This study |
| Δhu_xcc_-hu_xcc_ | iihu_xcc_ containing a pHM1:: hu_xcc_ vector, Rif^r^, Sp^r^ | This study |
| ΔhrpG-hrpG-his_6_ | ΔhrpG containing a pHM1::hrpG vector with the C terminal of HrpG addition of hexa-histidine tag, Rif^r^, Sp^r^ | This study |
| ΔhrpG-hrpG-HA-FLAG | ΔhrpG containing a pHM1::hrpG vector with the C terminal of HrpG addition of HA-Flag tag, Rif^r^, Sp^r^ | This study |
| **Plasmids** |  |  |
| pK18mob | *lacZα*, Kmr/mobilizable suicide vector, allows selection for insertion inactivation | Lab collection |
| pK18mobsacB | *sacB, lacZα*, Kmr/mobilizable *E. coli* suicide vector,  allows selection for double-crossover | Lab collection |
| pHM1 | Broad host range vector with pUC19 polylinker, Sp^r^ | Lab collection |
| pET30a | Protein expression vector; Kan^r^ | Lab collection |
| pET30a- hu_xcc_ | expressing full length HU_XCC_, Kan^r^ | This study |
| pQE30Xa | Protein expression vector; Amp^r^ | Lab collection |
| pQE30Xa-HrpG | expressing full length HrpG_Xcc_, Amp^r^ | This study |

^a^Rif^r^, Kan^r^, Ampr and Spc^r^ indicate resistance to rifampicin, kanamycin, ampicillin and spectinomycin, respectively.
