# Supplementary material for: Analysis of HrpG regulons and HrpG‐interacting proteins by ChIP‐seq and affinity proteomics in Xanthomonas campestris
Source: Mol Plant Pathol. 2020 Jan 8;21(3):388–400. doi: 10.1111/mpp.12903 (PMC7036363; doi:10.1111/mpp.12903)
Supplement: Supplementary file 4 — Table S2 Primers used in this study. [file MPP-21-388-s004.docx]

**Table S2.** Primers used in this study.

| Primer | Sequence (5’→3’) | Purpose |
| --- | --- | --- |
| △hrpG-up-F | TCCCCCGGGGTAAGAGCTGTGTTGCGGCTAAC | 504-bp DNA sequence upstream of *hrpG*, used for construction of *hrpG* deletion mutant. |
| △hrpG-up-R | AACTGCAGCACAATCAGTGGGGTGGGG |  |
| △hrpG-down-F | AACTGCAGCTGTTTTCGAATCCGGGCGTG | 549-bp DNA sequence downstream of *hrpG*, used for construction of *hrpG* deletion mutant. |
| △hrpG-down-R | CCCAAGCTTCAATATTTCCGGTGTCGGCGATG |  |
| ChrpG-F | CCCAAGCTTGTGATGGACGCCGCTGC | DNA fragment of 806-bp *hrpG* coding sequence, used for complementation, overproduction and site-directed mutagenesis of *hrpG*. |
| ChrpG-R | CGGAATTCTCAGCAAGCTGCGGTGCG |  |
| PhrpG-F | ATAGGATCCATGGACGCCGCTGCGAATG | DNA fragment encoding HrpG fused with 6×his_6_-tag N-terminally, used for constructing *Xcc* strain producing 6×his_6_::HrpG protein. |
| PhrpG-R | GCGAAGCTTTTAGCAAGCTGCGGTGCG |  |
| HrpG-his_6_-F | AAGCTTGTGATGGACGCCGCTGCG | DNA fragment encoding HrpG fused with 6×his_6_-tag C-terminally, used for constructing *Xcc* strain for ChIP-seq |
| HrpG-his_6_-R | GAATTCTCAGTGGTGGTGGTGGTGGTGGCAAGCTGCGGTGCGATT |  |
| HrpG-HA-Flag-F | AAGCTTGTGATGGACGCCGCTGCG | DNA fragment encoding HrpG fused with HA-Flag-tag C-terminally, used for constructing *Xcc* strain for TAP |
| HrpG-HA-Flag-R | GAATTCTCACTTGTCGTCGTCGTCCTTGTAGTCGACCTTGAGAGCGTAATCTGGAACATCGTATGGGTAGCAAGCTGCGGTGCGATT |  |
| △hu_xcc_-F | GGATCCTTGCCGCTGCTGCTGACAT | 234-bp DNA sequence used for construction of XC3262 insertional mutant. |
| △hu_xcc_-R | AAGCTTCATCCTTCAGGGCTTTGCC |  |
| Chu_xcc_-F | AAGCTTATGAATAAAACCGAATTGATC | DNA fragment of 273-bp XC3262 coding sequence, used for complementation, overproduction and site-directed mutagenesis of XC3262. |
| Chu_xcc_-R | GGTACCCTGAAGGATGCAGTAAACTAA |  |
| Phu_xcc_-F | CATATGAATAAAACCGAATTGATCG | DNA fragment encoding XC3262 fused with 6×his_6_-tag N-terminally, used for constructing *Xcc* strain producing 6×his::3262 protein. |
| Phu_xcc_-R | AAGCTTCCCTGAAGGATGCAGTAAAC |  |
| △hpa2-up-F | GAATTCTGGATCAATCGAGGTGTGCGTC | 309-bp DNA sequence upstream of XC3001, used for construction of XC3001 deletion mutant. |
| △hpa2-up-R | GGATCCTGGATTGACATGTTGATAGCCG |  |
| △hpa2-down-F | GGATCCGCTGTGGGTGCCTATCATTCC | 294-bp DNA sequence downstream of XC3001, used for construction of XC3001 deletion mutant. |
| △hpa2-down-R | AAGCTTGCTCGCCAACACGGTGTCC |  |
| Chpa2-F | AAGCTTTTGATTGCGGCGGCGTTGC | DNA fragment of 417-bp XC3001 coding sequence, used for complementation, overproduction and site-directed mutagenesis of XC3001. |
| Chpa2-R | GGTACCTCATTCGACAACCACACCGCGC |  |
| △hrcU-up-F | GAATTCAACCGGGACATTCAATCTTTTC | 372-bp DNA sequence upstream of XC3012, used for construction of XC3012 deletion mutant. |
| △hrcU-up-R | GGATCCTGCCAGCTTCATCACCAGC |  |
| △hrcU-down-F | GGATCCCTGCATGAGGTCGAACTGGG | 351-bp DNA sequence downstream of XC3012, used for construction of XC3012 deletion mutant. |
| △hrcU-down-R | AAGCTTTGGTGAACAGCAGCAACGAG |  |
| ChrcU-F | AAGCTTATGTCGGATGAGAAGACCGAGAAG | DNA fragment of 1074-bp XC3012 coding sequence, used for complementation, overproduction and site-directed mutagenesis of XC3012. |
| ChrcU-R | GGTACCCTAGCAGGGCAGCGGACCG |  |
| △hrpE-up-F | GAATTCACCGACACGCTCACCCAGG | 343-bp DNA sequence upstream of XC3021, used for construction of XC3021 deletion mutant. |
| △hrpE-up-R | GGATCCTCGCGGCACTATCGATTGTAA |  |
| △hrpE-down-F | GGATCCAACAAATTCATCGGCAAGGCA | 270-bp DNA sequence downstream of XC3021, used for construction of *XC3021* deletion mutant. |
| △hrpE-down-R | AAGCTTACTTGCTGTGCTTCATCGTCGG |  |
| ChrpE-F | AAGCTTATGCTAAATTTACAATCGATAGTGCCG | DNA fragment of 273-bp XC3021 coding sequence, used for complementation, overproduction and site-directed mutagenesis of XC3021. |
| ChrpE-R | GGTACCTTACTGGCCAACCAGCTGCTTC |  |
| 0124-EMSA-F | CACCAACGAAGCCTCCGACG | DNA fragment of XC0124 promoter spanning nucleotides from -290 to -6 relative to TIS, used for EMSA. |
| 0124-EMSA-R | GGCTCTCTCCAGGGCACTGG |  |
| 0125-EMSA-F | ACATCAACAACATCCTCGCCCA | DNA fragment of XC0125 promoter spanning nucleotides from -279 to -5 relative to TIS, used for EMSA. |
| 0125-EMSA-R | GTTCTCCTGTCAGAACTTGTAGCGC |  |
| 0126-EMSA-F | GTACGCCAGCGTGAGTTTCATCA | DNA fragment of XC0126 promoter spanning nucleotides from -231 to -14 relative to TIS, used for EMSA. |
| 0126-EMSA-R | AGGAGCCCAGCACGGTGGA |  |
| 1049-EMSA-F | TAGGTGTCCGGCAGTGGCG | DNA fragment of XC1049 promoter spanning nucleotides from -289 to -19 relative to TIS, used for EMSA. |
| 1049-EMSA-R | GAGTCACTGACGCGGCGGTT |  |
| 1050-EMSA-F | GTGGTGGACAAGCCGTTCG | DNA fragment of XC1050 promoter spanning nucleotides from -250 to -8 relative to TIS, used for EMSA. |
| 1050-EMSA-R | TCCACCAGCTGGTTTTCCAC |  |
| 1. EMSA-F 2. EMSA-R | CAGGTACAGCGTGAACGCG  AATGTGAAAGATTGAATGACAAGTGA | DNA fragment of XC1164 promoter spanning nucleotides from -257 to -11 relative to TIS, used for EMSA. |
| 1298-EMSA-F | AGCATGCAGCAATCACACGC | DNA fragment of XC1298 promoter spanning nucleotides from -249 to -16 relative to TIS, used for EMSA. |
| 1298-EMSA-R | GGAATTCGAAAACGCTGCAGG |  |
| 2579-EMSA-F | TCAGCAAAGAACGCGACACCT | DNA fragment of XC2579 promoter spanning nucleotides from -260 to -8 relative to TIS, used for EMSA. |
| 2579-EMSA-R | ACACTTCGCCGATAAACACCTCTA |  |
| 3001-EMSA-F | CCGTTATGGTGCTGCGATC | DNA fragment of XC3001 promoter spanning nucleotides from -252 to -5 relative to TIS, used for EMSA. |
| 3001-EMSA-R | CGTCGCAGAGGGGCAGCA |  |
| 3012-EMSA-F | CAGCCCGCTCACGACCGAA | DNA fragment of XC3012 promoter spanning nucleotides from -254 to -11 relative to TIS, used for EMSA. |
| 3012-EMSA-R | GGCCGCTATTGCTGCACCC |  |
| 3015-EMSA-F | TCGCCAGACCAGCTATCGCTT | DNA fragment of XC3015 promoter spanning nucleotides from -273 to -31 relative to TIS, used for EMSA. |
| 3015-EMSA-R | GTGGGTCAGCGCCTGCGA |  |
| 3017-EMSA-F | CTGGCACCGGCATTCACG | DNA fragment of XC3017 promoter spanning nucleotides from -250 to -18 relative to TIS, used for EMSA. |
| 3017-EMSA-R | TGGCGTCACCGATAACTCAGG |  |
| 3021-EMSA-F | GACACGCTCACCCAGGATATGA | DNA fragment of XC3021 promoter spanning nucleotides from -310 to -18 relative to TIS, used for EMSA. |
| 3021-EMSA-R | GAAGAGAAGTAACGTGGAGCGC |  |
| 3076-EMSA-F | GGATTAACGGCCATTTGACATTG | DNA fragment of XC3076 promoter spanning nucleotides from -320 to -11 relative to TIS, used for EMSA. |
| 3076-EMSA-R | CTGAAGGGGAGGAGCAAAAACAA |  |
| 3263-EMSA-F | ATCGGTGCTGCTGGATACGATG | DNA fragment of XC3263 promoter spanning nucleotides from -295 to -15 relative to TIS, used for EMSA. |
| 3263-EMSA-R | GATCGAGGAATTGGGGCGTAA |  |
| 1050-qRT-F | AGTGGTACCAGCTACGCGATGG | 237-bp DNA fragment spans nucleotides from 137 to 354 bp of the XC1050, used for RT-PCR. |
| 1050-qRT-R | GCATGACCTTGGATGCGGAC |  |
| 1298-qRT-F | GGCAAGTTCGACTTCGGCA | 237-bp DNA fragment spans nucleotides from 202 to 419 bp of the XC1298, used for RT-PCR. |
| 1298-qRT-R | ACTGGAGTTGCCTTCCAGCG |  |
| 3001-qRT-F | CATGGGTGCTACGCGCC | 255-bp DNA fragment spans nucleotides from 86 to 319 bp of the XC3001, used for RT-PCR. |
| 3001-qRT-R | CGGAATGATAGGCACCCACAGC |  |
| 3012-qRT-F | ATCGGCATCCAGGGTGTCTTG | 206-bp DNA fragment spans nucleotides from 238 to 424 bp of the XC3012, used for RT-PCR. |
| 3012-qRT-R | GCCTTGATGCCCAGCTTCAG |  |
| 3021-qRT-F | GATAGTGCCGCGATTGGGC | 238-bp DNA fragment spans nucleotides from 18 to 238 bp of the XC3021, used for RT-PCR. |
| 3021-qRT-R | CTTCGCGTTGTCGCCTGC |  |
| 3076-qRT-F | GCCGATGCCTTGTTGATTCTG | 262-bp DNA fragment spans nucleotides from 184 to 427 bp of the XC3076, used for RT-PCR. |
| 3076-qRT-R | TGCTCTGGCCCAGGTGGAA |  |
| 3262-qRT-F | TTGCCGCTGCTGCTGACAT | 167-bp DNA fragment spans nucleotides from 29 to 178 bp of the XC3262, used for RT-PCR. |
| 3262-qRT-R | CGTCTTCGGATTGCGGCC |  |
| Motif-1-F | CTTCGAAGTGAGAAATTCTATTGGATCCGC | 30-bp DNA fragment spans nucleotides from -103 to -74 bp of the XC3076, used for MST. |
| Motif-1-R | GCGGATCCAATAGAATTTCTCACTTCGAAG |  |
| Motif-2-F | CTTCGAAGTGAGAACGGAATCCGGATCCGC | Key base mutations of Motif 1, used for MST. |
| Motif-2-R | GCGGATCCGGATTCCGTTCTCACTTCGAAG |  |

The underlined sequences indicate the restriction sites for *Bam*HI, *Eco*RI, *Hin*dIII, *NdeI*, *PstI*, *SmaI*,and *Xba*I, respectively.
