# Supplementary material for: Analysis of HrpG regulons and HrpG‐interacting proteins by ChIP‐seq and affinity proteomics in Xanthomonas campestris
Source: Mol Plant Pathol. 2020 Jan 8;21(3):388–400. doi: 10.1111/mpp.12903 (PMC7036363; doi:10.1111/mpp.12903)
Supplement: Supplementary file 5 — Table S3 ChIP‐seq analysis revealed HrpG regulated genes of Xanthomonas campestris pv. campestris grown in the XCM2 inducing medium. [file MPP-21-388-s005.doc]

**Table S3.** ChIP-seq analysis revealed HrpG regulated genes of *Xanthomonas campestris* pv. *campestris* grown in the XCM2 inducing medium.

| **Gene ID** | **Annotation** | **Peak Z-score** | **Peak start:end** | **Summit** | **Strand** | **Region** |
| --- | --- | --- | --- | --- | --- | --- |
| 1. **Transport protein** | | | | | | |
| XC_0627 | D-serine/D-alanine/glycine transporter | 22.91327 | 367742:368042 | 367892 | + | u |
| XC_4125 | cation transporter, cation efflux system protein (czcA) | 22.36808 | 3596566:3596866 | 3596716 | + | u |
| XC_0310 | preprotein translocase subunit TatD | 20.44773 | 2032890:2033190 | 2033040 | + | ui |
| XC_1041 | conjugal transfer protein | 18.31684 | 746336:746636 | 746486 | + | u |
| XC_3786 | ABC transporter permease | 16.84627 | 1257822:1258122 | 1257972 | + | u |
| XC_4212 | TonB-dependent receptor | 16.32673 | 4977343:4977643 | 4977493 | + | u |
| XC_3474 | TonB-dependent receptor | 14.85541 | 4977343:4977643 | 4977493 | + | u |
| XC_2322 | chemoreceptor glutamine deamidase CheD | 13.24139 | 5109293:5109593 | 5109443 | + | u |
| XC_0919 | TonB-dependent receptor | 12.22631 | 2805996:2806296 | 2806146 | + | ui |
| XC_1284 | TonB-dependent receptor | 10.414 | 1101139:1101439 | 1101289 | + | ui |
| XC_0305 | putative MFS transporter, xanthine/uracil permease | 10.25274 | 1568979:1569279 | 1569129 | + | ui |
| XC_4058 | sodium ABC transporter permease | 8.75898 | 691209:691509 | 691359 | + | u |
| XC_2712 | phosphate transport system protein (phoU) | 8.78572 | 3257627:3257927 | 3257777 | + | ui |
| XC_0361 | MFS transporter | 8.38609 | 4779125:4779425 | 4779275 | + | u |
| XC_1644 | TonB-dependent receptor | 8.08588 | 419961:420261 | 420111 | + | ui |
| XC_0780 | EamA/RhaT family transporter | 7.76797 | 936945:937245 | 937095 | + | u |
| XC_0737 | TonB-dependent receptor | 7.42048 | 1975780:1976080 | 1975930 | + | u |
| XC_1693 | Fe^2+^-trafficking protein | 6.715 | 93186:93486 | 93336 | + | u |
| XC_3459 | ABC transporter permease, permease (nrtB) | 6.39093 | 4107499:4107799 | 4107649 | + | u |
| XC_0084 | proline/glycine betaine transporter ProP | 5.92054 | 98619:98919 | 98769 | + | u |
| XC_0810 | citrate transporter | 5.70697 | 970315:970615 | 970465 | + | u |
| XC_3730 | sugar ABC transporter permease | 5.55793 | 4416160:4416460 | 4416310 | + | u |
| XC_1712 | TonB-dependent receptor | 5.54226 | 2059741:2060041 | 2059891 | + | ui |
| XC_0124 | TonB-dependent receptor | 5.06279 | 149382:149682 | 149532 | + | u |
| XC_1434 | multidrug transporter | 4.95372 | 1731465:1731765 | 1731615 | + | ui |
| XC_0174 | amino acid ABC transporter permease | 3.73807 | 216381:216681 | 216531 | + | u |
| XC_2453 | MFS transporter, Prop transport protein (proP) | 3.57586 | 2965443:2965743 | 2965593 | + | ui |
| XC_2143 | MFS transporter | 3.25337 | 2570480:2570780 | 2570630 | + | u |
| 1. **Signal transduction** | | | | | | |
| XC_1050 | sensor histidine kinase | 155.5969 | 1265708:1266008 | 1265858 | + | u |
| XC_1526 | two-component system sensor histidine kinase | 23.12241 | 1836957:1837257 | 1837107 | + | ui |
| XC_2579 | histidine kinase, response regulator hybrid protein | 14.26106 | 3115158:3115458 | 3115308 | + | ui |
| XC_0813 | sensor histidine kinase | 9.49489 | 972194:972494 | 972344 | + | ui |
| XC_1150 | response regulator | 7.0449 | 1389743:1390043 | 1389893 | + | ui |
| XC_0635 | histidine kinase | 6.476 | 757138:757438 | 757288 | + | ui |
| XC_3982 | HAMP domain-containing protein, two-component system, sensor protein (ygiY) | 5.32229 | 4698198:4698498 | 4698348 | + | u |
| XC_2252 | response regulator | 4.34235 | 4902635:4902935 | 4902785 | + | ui |
| XC_0818 | sensor histidine kinase | 1.4454 | 979115:979415 | 979265 | + | ui |
| 1. **Transcription and translation** | | | | | | |
| XC_0559 | peptidase | 20.99483 | 669355:669655 | 669505 | + | u |
| XC_0438 | RNA helicase | 14.01261 | 521269:521569 | 521419 | + | ui |
| XC_3949 | acyltransferase | 13.26994 | 5096735:5097035 | 5096885 | + | ui |
| XC_0097 | TldD | 10.95375 | 1151136:115436 | 115286 | + | ui |
| XC_0643 | aminopeptidase | 10.82864 | 772944:773244 | 773094 | + | u |
| XC_4009 | peptidase M20 | 10.19814 | 4727846:4728146 | 4727996 | + | ui |
| XC_0096 | TldD | 5.48036 | 113139:113439 | 113289 | + | u |
| 1. **Cell structure and division** | | | | | | |
| XC_4211 | membrane protein, putative hexuronate transporter (exuT) | 16.32673 | 4481064:4481364 | 4481214 | + | u |
| XC_1584 | group 1 truncated hemoglobin, cyanoglobin | 14.42803 | 1904122:1904422 | 1904272 | + | u |
| XC_4308 | membrane protein | 13.51899 | 4128630:4128930 | 4128780 | + | ui |
| XC_0950 | porin | 9.30446 | 1142331:1142631 | 1142481 | + | u |
| XC_3960 | membrane protein, outer membrane lipoprotein Blc (blc) | 5.7677 | 4677895:4678195 | 4678045 | + | u |
| XC_2152 | membrane protein | 5.03334 | 2581504:2581804 | 2581654 | + | u |
| XC_1237 | membrane protein | 4.61328 | 1512681:1512981 | 1512831 | + | ui |
| XC_3783 | membrane protein | 4.21004 | 4475579:4475879 | 4475729 | + | ui |
| 1. **Cellular processes** | | | | | | |
| XC_0519 | ice nucleation protein | 20.31201 | 1671890:1672190 | 1672040 | + | ui |
| XC_4292 | microcystin dependent protein | 18.58711 | 612227:612527 | 612377 | + | ui |
| XC_3058 | chemotaxis protein CheR | 7.1228 | 881605:881905 | 881755 | + | u |
| XC_1201 | RebB protein | 7.1995 | 4396035:4396335 | 4396185 | + | ui |
| XC_2316 | chemotaxis protein (tsr) | 6.45644 | 2779762:2780062 | 2779912 | + | u |
| 1. **Amino acids metabolism** | | | | | | |
| XC_0481 | type 1 glutamine amidotransferase | 60.19013 | 571256:571556 | 571406 | + | ui |
| XC_0473 | anthranilate synthase component I | 45.48382 | 562272:562572 | 562422 | + | ui |
| XC_3588 | valine--tRNA ligase | 29.1261 | 4265964:4266264 | 4266114 | + | u |
| XC_2560 | aminotransferase | 13.19545 | 3095761:3096061 | 3095911 | + | ui |
| XC_1155 | N-acetyltransferase | 8.5933 | 1396803:1397103 | 1396953 | + | u |
| XC_2724 | 5-methyltetrahydrofolate--homocysteine methyltransferase | 7.87911 | 3273198:3273498 | 3273348 | + | ui |
| XC_0721 | SAM-dependent methyltransferase | 7.38153 | 865382:865682 | 865532 | + | u |
| XC_3401 | glutamine amidotransferase | 6.71978 | 4053642:4053942 | 4053792 | + | u |
| XC_1191 | alanine acetyltransferase | 4.87586 | 1446694:1446994 | 1446844 | + | u |
| XC_0882 | aspartate aminotransferase family protein | 4.07957 | 1062205:1062505 | 1062355 | + | u |
| 1. **Energy metabolism** | | | | | | |
| XC_0436 | short-chain dehydrogenase | 18.80003 | 518114:518414 | 518264 | + | ui |
| XC_1452 | oxidoreductase | 15.22406 | 1750938:1751238 | 1751088 | + | ui |
| XC_2132 | glyoxalase/bleomycin resistance/dioxygenase family protein | 12.23632 | 2558351:2558651 | 2558501 | + | ui |
| XC_3138 | aldo/keto reductase, oxidoreductase | 10.35193 | 3749961:3750261 | 3750111 | + | ui |
| XC_0396 | 3-oxoacyl-ACP, oxidoreductase | 9.94777 | 459218: 459518 | 459368 | + | ui |
| XC_3901 | cytochrome C oxidase subunit III | 9.70057 | 4606886:4607186 | 4607036 | + | u |
| XC_3707 | oxidoreductase | 9.0574 | 4393858:4394158 | 4394008 | + | ui |
| XC_0705 | endopolygalacturonase | 8.64449 | 4862453:4862753 | 4862603 | + | ui |
| XC_0887 | cytochrome C6 | 7.76631 | 1069004:1069304 | 1069154 | + | ui |
| XC_3490 | phosphoglycerate mutase | 7.64261 | 4146615:4146915 | 4146765 | + | ui |
| XC_0890 | azurin | 7.6034 | 1071539:1071839 | 1071689 | + | u |
| XC_0913 | flavin reductase | 6.1265 | 1094428:1094728 | 1094578 | + | ui |
| XC_3774 | alcohol dehydrogenase(Zn-dependent) | 5.80821 | 4465269:4465569 | 4465419 | + | ui |
| XC_3740 | aldo/keto reductase | 5.15133 | 4426942:4427242 | 4427092 | + | ui |
| XC_3493 | NAD(P)H quinone oxidoreductase | 3.49252 | 4151469:4151769 | 4151619 | + | ui |
| XC_1384 | alcohol dehydrogenase | 3.20621 | 1671890:1672190 | 1672040 | + | u |
| 1. **Fatty acid and phospholipid acid metabolism** | | | | | | |
| XC_0429 | 3-oxoacyl-ACP reductase | 30.73509 | 508857:509157 | 509007 | + | u |
| XC_0387 | lipase | 21.77062 | 448357:448657 | 448507 | + | u |
| XC_0254 | 3-methylcrotonyl-CoA | 13.92683 | 309982:310282 | 310132 | + | ui |
| XC_3424 | acyl-CoA synthetase | 11.55578 | 4073062:4073362 | 4073212 | + | u |
| XC_0229 | 3-beta hydroxysteroid | 10.28171 | 277489:277789 | 277639 | + | u |
| XC_0151 | lysophospholipase | 8.8808 | 190117:190417 | 190267 | + | u |
| XC_0573 | malonate decarboxylase ACP | 8.86488 | 685951:686251 | 686101 | + | u |
| XC_0917 | acetyl-CoA hydrolase | 5.97316 | 1097902:1098202 | 1098052 | + | ui |
| XC_0039 | acyl-ACP phosphodiesterase | 2.78085 | 49665:49965 | 49815 | + | ui |
| 1. **Nucleotide metabolism** | | | | | | |
| XC_0518 | phosphatidate cytidylyltransferase | 30.08739 | 610448:610748 | 610598 | + | ui |
| XC_0322 | formyltetrahydrofolate deformylase | 14.95554 | 380493:380793 | 380643 | + | ui |
| XC_3791 | enamine deaminase RidA | 6.6768 | 4485968:4486268 | 4486118 | + | ui |
| XC_0480 | type I restriction-modification system subunit M, type I site-specific deoxyribonuclease | 19.97187 | 568951:569251 | 569101 | + | u |
| XC_1389 | nucleotidyltransferase family protein | 13.54943 | 1678640:1678940 | 1678790 | + | u |
| XC_0633 | endonuclease | 9.86603 | 753617:753917 | 753767 | + | ui |
| XC_1540 | methylated-DNA--protein-cysteine methyltransferase | 8.0781 | 1847810:1848110 | 1847960 | + | u |
| XC_0235 | pseudouridylate synthase | 6.31179 | 282672:282972 | 282822 | + | u |
| XC_2795 | diguanylate cyclase | 3.5512 | 3409911:3410211 | 3410061 | + | u |
| 1. **Biosynthesis of small molecules** | | | | | | |
| XC_0576 | phosphoribosyl-dephospho-CoA transferase | 15.23314 | 687512:687812 | 687662 | + | ui |
| XC_4225 | tryptophan halogenase | 12.5737 | 4999264:4999564 | 4999414 | + | ui |
| XC_0835 | class I SAM-dependent methyltransferase | 10.87057 | 1005449:1005749 | 1005599 | + | ui |
| XC_3062 | thioredoxin TrxC | 9.58532 | 3664896:3556196 | 3665046 | + | ui |
| XC_1094 | | 23S rRNA pseudouridine 2605 synthase | | --- | | 4.47684 | 1320417:1320717 | 1320567 | + | u |
| XC_1095 | segregation and condensation protein B | 4.47684 | 1320417:1320717 | 1320567 | + | ui |
| XC_0798 | methyltransferase | 2.98492 | 953842:954142 | 953992 | + | ui |
| 1. **Central intermediary metabolism** | | | | | | |
| XC_1642 | alpha-glucosidase | 265.91611 | 1967545:1967845 | 1967695 | + | u |
| XC_0682 | methanol dehydrogenase | 40.29854 | 816676:816976 | 816826 | + | ui |
| XC_2991 | beta-glucosidase (bglX) | 30.32658 | 3586712:3587012 | 3586862 | + | u |
| XC_0163 | rhamnogalacturonan | 21.40887 | 203496:203796 | 203646 | + | ui |
| XC_0384 | alpha/beta hydrolase | 19.01049 | 445189:445489 | 445339 | + | ui |
| XC_0382 | 3-oxoadipate enol-lactonase | 19.01049 | 445189:445489 | 445339 | + | u |
| XC_0048 | saccharopine dehydrogenase | 16.4905 | 58907:59207 | 59057 | + | ui |
| XC_0679 | methanol dehydrogenase heavy chain | 15.40884 | 814670:814970 | 814820 | + | u |
| XC_3708 | hydrolase | 12.39756 | 4663346:4663646 | 4663496 | + | u |
| XC_0603 | esterase | 10.22204 | 721337:721637 | 721487 | + | ui |
| XC_2588 | glycosyl transferase | 9.85651 | 3120943:3121243 | 3121093 | + | ui |
| XC_3416 | gluconolactonase | 9.04183 | 4066826:4067126 | 4066976 | + | ui |
| XC_0581 | haloacid dehalogenase | 8.6055 | 837749:838049 | 837899 | + | u |
| XC_0428 | NAD-dependent dehydratase | 7.86618 | 506378:506678 | 506528 | + | ui |
| XC_0374 | diguanylate, phenoxybenzoate dioxygenase beta subunit | 7.16535 | 436637:436937 | 436787 | + | u |
| XC_4065 | beta-xylosidase | 6.83583 | 4783798:4784098 | 4783948 | + | u |
| XC_0176 | ergothioneine biosynthesis protein EgtB | 6.53251 | 219153:219453 | 219303 | + | ui |
| XC_4159 | L-fuconate dehydratase | 5.5158 | 1459144:1459444 | 1459294 | + | ui |
| XC_4196 | glycoside hydrolase family 43 protein, xylosidase/arabinosidase (xylB) | 5.05774 | 4954550:4954850 | 4954700 | + | u |
| XC_0461 | nucleoside-diphosphate sugar epimerase | 4.78766 | 547210:547510 | 547360 | + | ui |
| XC_1217 | glycoside hydrolase famiy 3 | 4.67314 | 846268:846568 | 846418 | + | ui |
| XC_0270 | alpha/beta hydrolase | 4.03118 | 324440:324740 | 324590 | + | u |
| XC_3618 | glycosyltransferase | 3.57586 | 4298241:4298541 | 4298391 | + | u |
| XC_2984 | arabinogalactan endo-1,4-beta-galactosidase | 3.44303 | 3573827:3574127 | 3573977 | + | ui |
| XC_0607 | glycosyl transferase | 3.19484 | 725337:725637 | 725487 | + | u |
| 1. **Mobile genetic elements** | | | | | | |
| XC_0137 | IS5-like element IS1478 family transposase | 64.59258 | 168966:169266 | 169116 | + | ui |
| XC_0681 | IS3 family transposase | 40.29854 | 816676:816976 | 816826 | + | u |
| XC_0134 | IS4 family transposase | 37.51445 | 163781:164081 | 163931 | + | ui |
| XC_2107 | replication initiation protein | 26.14721 | 2532142:2532442 | 2532292 | + | u |
| XC_0413 | IS3 family transposase | 24.77785 | 478929:479229 | 479079 | + | u |
| XC_1212 | IS3 family transposase | 23.73614 | 1473201:1473501 | 1473351 | + | u |
| XC_2113 | minor coat protein | 20.7606 | 2534438:2534738 | 2534588 | + | ui |
| XC_3625 | IS5/IS1182 family transposase | 11.98937 | 4304407:4304707 | 4304557 | + | ui |
| 1. **Pathogenicity, virulence, and adaptation** | | | | | | |
| XC_0699 | type VI secretion protein | 40.41405 | 1586178:1586478 | 1586328 | + | u |
| XC_1298 | pectate lyase | 18.13381 | 2028005:2028305 | 2028155 | + | u |
| XC_3001 | lytic transglycosylase, Hpa2 protein (hpa2) | 17.10012 | 153087:153387 | 153237 | + | ui |
| XC_1687 | general stress protein | 12.22331 | 153087:153387 | 153237 | + | u |
| XC_3012 | EscU/YscU/HrcU family type III secretion system export apparatus switch protein | 10.06244 | 3612691:3612991 | 3612841 | + | ui |
| XC_0125 | pectin methylesterase | 8.80984 | 1453121:1453421 | 1453271 | + | ui |
| XC_0126 | pectate lyase | 8.80984 | 3610256:3610556 | 3610406 | + | u |
| XC_3021 | HPr kinase, HrpE protein (hrpE) | 6.55437 | 775658:775958 | 775808 | + | ui |
| XC_1196 | copper homeostasis protein CutC | 4.11696 | 695030:695030 | 695180 | + | ui |
| XC_3017 | EscS/YscS/HrcS family type III secretion system export apparatus protein | 2.91041 | 1485299:1485599 | 1485449 | + | ui |
| 1. **Regulatory functions** | | | | | | |
| XC_0644 | transcriptional regulator | 17.49367 | 3605529:3605829 | 3605679 | + | u |
| XC_2253 | sigma-54-dependent Fis family transcriptional regulator, transcriptional regulator (fleQ) | 4.34235 | 2710843:2711143 | 2710993 | + | ui |
| XC_1383 | transcriptional regulator | 3.20621 | 2710843:2711143 | 2710993 | + | ui |
| 1. **Hypothetical proteins** | | | | | | |
| XC_2627 | hypothetical protein | 103.1521 | 3360623:3360923 | 3360773 | + | u |
| XC_1033 | hypothetical protein | 70.29108 | 3163322:3163622 | 3163472 | + | ui |
| XC_2997 | hypothetical protein | 45.01855 | 3592970:3593270 | 3593120 | + | ui |
| XC_0700 | hypothetical protein | 44.7765 | 1248239:1248539 | 1248389 | + | u |
| XC_0539 | hypothetical protein | 43.09438 | 840332:840632 | 840482 | + | u |
| XC_0656 | hypothetical protein | 42.10302 | 641623:641923 | 641773 | + | u |
| XC_4199 | hypothetical protein | 34.13764 | 789555:789855 | 789705 | + | ui |
| XC_1045 | hypothetical protein | 28.16589 | 4960641:4960941 | 4960791 | + | u |
| XC_0291 | hypothetical | 25.84728 | 1259279:1259579 | 1259429 | + | ui |
| XC_2474 | hypothetical protein | 24.6619 | 346374:346674 | 346524 | + | ui |
| XC_0103 | hypothetical protein | 23.21659 | 2990832:2991132 | 2990982 | + | u |
| XC_0071 | hypothetical protein | 19.36591 | 85620:85920 | 85770 | + | u |
| XC_0593 | hypothetical protein | 18.74887 | 121665:121965 | 121815 | + | u |
| XC_0560 | DUF3649 domain-containing protein | 17.69732 | 709568:709868 | 709718 | + | u |
| XC_0141 | hypothetical | 17.32725 | 174347:174647 | 174497 | + | ui |
| XC_0140 | hypothetical | 17.32725 | 670657:670957 | 670807 | + | u |
| XC_1711 | conserved hypothetical protein | 16.54245 | 174347:174647 | 174497 | + | u |
| XC_0720 | hypothetical protein | 12.40171 | 2056181:2056481 | 2056331 | + | u |
| XC_0105 | conserved hypothetical protein | 11.63077 | 864349:864649 | 864499 | + | ui |
| XC_4270 | hypothetical protein | 11.62128 | 124677:124977 | 124827 | + | ui |
| XC_0579 | hypothetical protein | 8.9364 | 363951:364251 | 364101 | + | ui |
| XC_3103 | hypothetical protein | 8.25664 | 5064682:5064982 | 5064832 | + | ui |
| XC_0178 | conserved hypothetical protein | 8.22445 | 3716352:3716652 | 3716502 | + | ui |
| XC_0794 | conserved hypothetical protein | 8.19386 | 220615:220915 | 220765 | + | u |
| XC_3863 | conserved hypothetical protein | 7.44897 | 949972:950272 | 950122 | + | u |
| XC_1083 | conserved hypothetical protein | 7.21234 | 4569029:4569329 | 4569179 | + | u |
| XC_0080 | conserved hypothetical protein | 7.0849 | 3661331:3661631 | 3661481 | + | ui |
| XC_0989 | conserved hypothetical protein | 5.56126 | 1306241:1306541 | 1306391 | + | u |
| XC_3984 | conserved hypothetical protein | 4.75123 | 1186475:1186775 | 1186625 | + | u |
| XC_1438 | conserved hypothetical protein | 4.03936 | 4699656:4699956 | 4699806 | + | ui |
| XC_3880 | conserved hypothetical protein | 3.85225 | 1736003:1736303 | 1736153 | + | ui |
| XC_2836 | conserved hypothetical protein | 3.69891 | 4584228:4584528 | 4584378 | + | ui |
