# Supplementary material for: Analysis of HrpG regulons and HrpG‐interacting proteins by ChIP‐seq and affinity proteomics in Xanthomonas campestris
Source: Mol Plant Pathol. 2020 Jan 8;21(3):388–400. doi: 10.1111/mpp.12903 (PMC7036363; doi:10.1111/mpp.12903)
Supplement: Supplementary file 6 — Table S4 Proteins identified by tandem affinity purification (TAP) under XCM2‐induced condition. [file MPP-21-388-s006.doc]

**Table S4.** Proteins identified by tandem affinity purification (TAP) under XCM2-induced condition.

| **Protein code** | **Annotation or description** | **Score** | **Theoretical MW(Da)** |
| --- | --- | --- | --- |
| 1. **Transport protein** | | | |
| XC_0009 | biopolymer transport ExbB protein | 18.39 | 26.6 |
| XC_1241 | TonB-dependent receptor | 13.63 | 100.9 |
| XC_1141 | TolB protein | 10.32 | 47.0 |
| XC_1116 | ABC transporter ATP-binding protein | 9.75 | 69.8 |
| XC_0849 | TonB-dependent receptor | 6.15 | 104.3 |
| XC_3546 | ABC transporter ATP-binding protein | 4.36 | 61.8 |
| XC_0011 | biopolymer transport ExbD2 protein | 4.36 | 15.0 |
| XC_3501 | preprotein translocase SecA subunit | 4.18 | 102.3 |
| XC_1345 | ABC transporter permease | 2.27 | 45.1 |
| XC_4299 | toluene tolerance protein | 2.00 | 23.5 |
| XC_0494 | bacterioferritin | 0.00 | 18.2 |
| XC_3666 | ABC transporter ATP-binding protein | 0.00 | 47.7 |
| XC_4066 | ABC transporter ATP-binding protein | 0.00 | 36.2 |
| 1. **Signal transduction** | | | |
| XC_2229 | histidine kinase | 9.48 | 45.7 |
| XC_3452 | two-component system regulatory protein | 8.04 | 28.1 |
| XC_0850 | response regulator | 3.93 | 39.6 |
| XC_4031 | two-component system regulatory protein | 2.33 | 25.4 |
| XC_3057 | two-component system sensor protein | 0.00 | 113.4 |
| 1. **Transcription and translation** | | | |
| XC_3342 | elongation factor Tu | 198.9 | 43.1 |
| XC_3346 | RNA polymerase beta' subunit | 114.70 | 155.2 |
| XC_3343 | elongation factor G | 96.42 | 77.6 |
| XC_3347 | RNA polymerase beta subunit | 73.10 | 154.8 |
| XC_2863 | 30S ribosomal protein S2 | 53.15 | 30.1 |
| XC_3349 | 50S ribosomal protein L10 | 27.59 | 18.3 |
| XC_3040 | 50S ribosomal protein L19 | 26.25 | 14.7 |
| XC_3316 | RNA polymerase alpha subunit | 18.88 | 36.3 |
| XC_3357 | 50S ribosomal protein L25 | 16.08 | 23.1 |
| XC_3317 | 30S ribosomal protein s4 | 14.84 | 23.3 |
| XC_1964 | transcription-related protein | 13.23 | 85.3 |
| XC_3339 | 50S ribosomal protein L4 | 11.65 | 21.8 |
| XC_3043 | 30S ribosomal protein S16 | 11.36 | 9.6 |
| XC_3328 | 50S ribosomal protein L5 | 9.81 | 20.1 |
| XC_3350 | 50S ribosomal protein L1 | 9.80 | 24.0 |
| XC_3340 | 50S ribosomal protein L3 | 9.00 | 22.9 |
| XC_2670 | 30S ribosomal protein S18 | 7.23 | 9.0 |
| XC_2864 | elongation factor Ts | 6.90 | 32.9 |
| XC_0490 | 50S ribosomal protein L13 | 6.60 | 16.1 |
| XC_3341 | 30S ribosomal protein S10 | 6.23 | 11.7 |
| XC_0945 | 50S ribosomal protein L31 | 6.18 | 9.3 |
| XC_3344 | 30S ribosomal protein S7 | 5.29 | 17.2 |
| XC_3334 | 30S ribosomal protein S3 | 5.10 | 27.4 |
| XC_1128 | aspartyl-tRNA synthetase | 4.87 | 64.8 |
| XC_2671 | 30S ribosomal protein S6 | 4.58 | 16.4 |
| XC_3324 | 50S ribosomal protein L18 | 4.49 | 12.8 |
| XC_3351 | 50S ribosomal protein L11 | 4.35 | 14.8 |
| XC_3806 | RNA polymerase sigma-70 factor | 4.35 | 69.9 |
| XC_2098 | ribonuclease E | 4.17 | 125.7 |
| XC_3330 | 50S ribosomal protein L14 | 4.13 | 13.5 |
| XC_3338 | 50S ribosomal protein L23 | 4.03 | 11.0 |
| XC_1608 | 30S ribosomal protein S15 | 4.02 | 10.0 |
| XC_3333 | 50S ribosomal protein L16 | 4.00 | 15.5 |
| XC_3945 | arginyl-tRNA synthetase | 3.95 | 61.9 |
| XC_3850 | transcription termination factor Rho | 3.89 | 47.8 |
| XC_3326 | 30S ribosomal protein S8 | 3.82 | 14.3 |
| XC_3348 | 50S ribosomal protein L7/L12 | 3.67 | 12.4 |
| XC_4123 | 50S ribosomal protein L28 | 3.65 | 9.1 |
| XC_3087 | isoleucyl-tRNA synthetase | 3.58 | 104.5 |
| XC_2669 | 50S ribosomal protein L9 | 3.47 | 15.7 |
| XC_1429 | glutamyl-tRNA synthetase | 2.28 | 52.0 |
| XC_3925 | tyrosyl-tRNA synthetase | 2.20 | 44.2 |
| XC_3352 | transcription antitermination factor | 2.18 | 21.2 |
| XC_1924 | 30S ribosomal protein S1 | 2.15 | 61.5 |
| XC_2900 | methionyl-tRNA synthetase | 1.94 | 75.5 |
| XC_1475 | ATP-dependent RNA helicase | 1.89 | 70.3 |
| XC_2507 | alanyl-tRNA synthetase | 1.76 | 95.2 |
| XC_1605 | protein chain initiation factor IF-2 | 1.72 | 97.5 |
| XC_3619 | acetyl transferase/isomerase | 1.67 | 33.2 |
| XC_1070 | ribosomal large subunit pseudouridine synthase D | 0.00 | 36.1 |
| XC_1249 | peptide chain release factor 3 | 0.00 | 58.9 |
| XC_1604 | N utilization substance protein A | 0.00 | 55.1 |
| XC_2673 | asparaginyl-tRNA synthetase | 0.00 | 51.9 |
| XC_3092 | 50S ribosomal protein L27 | 0.00 | 9.1 |
| XC_3309 | GTP-binding elongation factor protein | 0.00 | 62.4 |
| XC_3321 | 50S ribosomal protein L15 | 0.00 | 15.4 |
| XC_3409 | glutaminyl-tRNA synthetase | 0.00 | 65.6 |
| XC_3588 | valyl-tRNA synthetase | 0.00 | 106.0 |
| 1. **Cell structure and division** | | | |
| XC_3300 | outer membrane protein | 79.60 | 39.3 |
| XC_1142 | outer membrane protein P6 precursor | 23.27 | 18.4 |
| XC_0017 | outer membrane protein | 22.16 | 47.1 |
| XC_3694 | outer membrane protein | 11.88 | 23.5 |
| XC_3628 | membrane protein WxcE | 10.07 | 23.9 |
| XC_2944 | outer membrane protein | 6.38 | 10.7 |
| XC_1619 | Oar protein | 5.65 | 117.7 |
| XC_1058 | pilin | 3.59 | 14.8 |
| XC_2245 | flagellar protein | 2.07 | 40.6 |
| XC_0691 | rod shape-determining protein | 1.85 | 37.4 |
| XC_0828 | TolC protein | 1.61 | 49.4 |
| XC_0941 | fimbrial assembly membrane protein | 0.00 | 38.3 |
| 1. **Cellular processes** | | | |
| XC_3617 | aminotransferase | 7.11 | 38.8 |
| XC_3122 | septum site-determining protein | 5.60 | 28.9 |
| XC_3630 | glycosyltransferase | 4.42 | 39.9 |
| XC_3615 | electron transfer flavoprotein alpha subunit | 0.00 | 31.8 |
| 1. **Amino acids metabolism** | | | |
| XC_3135 | glycine decarboxylase | 13.77 | 104.7 |
| XC_3290 | threonine 3-dehydrogenase | 7.23 | 37.0 |
| XC_4015 | arginase | 6.87 | 33.1 |
| XC_3296 | 2-amino-3-ketobutyrate CoA ligase | 5.22 | 43.5 |
| XC_0100 | aromatic-amino-acid aminotransferase | 4.82 | 43.0 |
| XC_1367 | aspartate carbamoyltransferase | 4.81 | 33.8 |
| XC_0194 | glutamine synthetase | 4.60 | 51.8 |
| XC_0836 | 3-isopropylmalate dehydrogenase | 4.30 | 38.0 |
| XC_1569 | aspartate semialdehyde dehydrogenase | 2.21 | 36.6 |
| XC_0834 | 3-isopropylmalate dehydratase small subunit | 2.07 | 24.2 |
| XC_2351 | valine-pyruvate aminotransferase | 2.07 | 44.5 |
| XC_0487 | S-adenosyl methionine decarboxylase proenzyme | 2.00 | 30.8 |
| XC_4041 | shikimate 5-dehydrogenase | 1.99 | 29.7 |
| XC_3313 | family II 2-keto-3-deoxy-D-arabino-heptulosonate 7-phosphate synthase | 1.86 | 53.3 |
| XC_3954 | spermidine synthase | 1.69 | 31.5 |
| XC_3544 | serine hydroxymethyltransferase | 1.61 | 44.8 |
| 1. **Energy metabolism** | | | |
| XC_3627 | putative GDP-mannose 4,6-dehydratase | 80.54 | 38.7 |
| XC_3678 | ATP synthase beta chain | 43.20 | 51.0 |
| XC_1063 | succinyl-CoA synthetase beta subunit | 39.54 | 41.4 |
| XC_0979 | fructose-bisphosphate aldolase | 33.72 | 36.3 |
| XC_2750 | dihydrolipoamide S-succinyltransferase | 32.84 | 42.4 |
| XC_0944 | citrate synthase | 27.11 | 47.7 |
| XC_0588 | pyruvate dehydrogenase | 26.94 | 99.7 |
| XC_1595 | NADH-ubiquinone oxidoreductase NQO3 subunit | 26.86 | 79.0 |
| XC_2531 | enolase | 26.07 | 45.9 |
| XC_3691 | dihydrolipoamide acetyltranferase | 18.87 | 59.8 |
| XC_3680 | ATP synthase alpha chain | 18.15 | 55.3 |
| XC_1064 | succinyl-CoA synthetase alpha subunit | 14.86 | 29.6 |
| XC_0972 | glyceraldehyde-3-phosphate dehydrogenase | 13.64 | 36.0 |
| XC_2749 | oxoglutarate dehydrogenase | 12.76 | 109.3 |
| XC_0690 | sugar kinase | 12.45 | 34.6 |
| XC_0441 | dihydrolipoamide acyltransferase | 9.00 | 51.6 |
| XC_0978 | pyruvate kinase type II | 8.64 | 54.5 |
| XC_1592 | NADH-ubiquinone oxidoreductase NQO4 subunit | 8.34 | 49.4 |
| XC_2751 | dihydrolipoamide dehydrogenase | 8.24 | 50.2 |
| XC_2326 | aconitate hydratase 2 | 6.59 | 92.8 |
| XC_3854 | isocitrate dehydrogenase | 5.73 | 80.0 |
| XC_3268 | isocitrate dehydrogenase | 5.45 | 35.5 |
| XC_3689 | dihydrolipoamide dehydrogenase | 4.72 | 21.3 |
| XC_2822 | fumarate hydratase | 4.21 | 55.8 |
| XC_0976 | phosphoglycerate kinase | 3.64 | 40.7 |
| XC_3396 | alkyl hydroperoxide reductase subunit C | 3.61 | 20.4 |
| XC_1594 | NADH-ubiquinone oxidoreductase NQO1 subunit | 3.53 | 48.1 |
| XC_1404 | phosphoglycerate mutase | 3.47 | 27.9 |
| XC_1593 | NADH-ubiquinone oxidoreductase NQO2 subunit | 2.39 | 19.5 |
| XC_1837 | superoxide dismutase | 1.77 | 22.7 |
| XC_1591 | NADH-ubiquinone oxidoreductase NQO5 subunit | 1.74 | 27.9 |
| XC_2824 | ferredoxin-NADP reductase | 1.66 | 29.4 |
| XC_0104 | chloroacetaldehyde dehydrogenase | 0.00 | 55.5 |
| XC_0281 | oxidoreductase | 0.00 | 34.4 |
| XC_0761 | betaine aldehyde dehydrogenase | 0.00 | 52.5 |
| XC_0726 | oxidoreductase | 0.00 | 38.5 |
| XC_1590 | NADH-ubiquinone oxidoreductase NQO6 subunit | 0.00 | 21.1 |
| XC_1985 | succinate dehydrogenase iron-sulfur protein | 0.00 | 29.3 |
| 1. **Fatty acid and phospholipid acid metabolism** | | | |
| XC_3652 | beta-ketoacyl-[ACP] synthase I | 12.24 | 41.9 |
| XC_4149 | acetyl coenzyme A synthetase | 4.38 | 71.4 |
| XC_3226 | acyl carrier protein | 4.13 | 8.8 |
| XC_2943 | acetoacetyl-CoA thiolase | 4.12 | 39.8 |
| XC_0527 | biotin carboxylase subunit of acetyl CoA carboxylase | 1.60 | 49.3 |
| XC_3225 | 3-oxoacyl-[ACP] synthase II | 0.00 | 43.0 |
| 1. **Nucleotide metabolism** | | | |
| XC_2346 | carbamoyl-phosphate synthase large chain | 29.34 | 117.2 |
| XC_2509 | RecA protein | 15.92 | 37.0 |
| XC_1378 | single-stranded DNA binding protein | 14.39 | 18.6 |
| XC_0004 | DNA gyrase subunit B | 8.54 | 89.4 |
| XC_3262 | histone-like protein | 8.30 | 9.3 |
| XC_1548 | deoxycytidine triphosphate deaminase | 6.43 | 21.3 |
| XC_2534 | CTP synthetase | 6.16 | 62.5 |
| XC_1163 | DNA-binding related protein | 5.89 | 20.4 |
| XC_4070 | 2-dehydropantoate 2-reductase | 4.27 | 32.8 |
| XC_1934 | inosine-5'-monophosphate dehydrogenase | 3.55 | 51.5 |
| XC_1324 | phosphoribosylformylglycinamide cyclo-ligase | 3.42 | 35.8 |
| XC_3578 | phosphoribosylformylglycinamidine synthetase | 2.06 | 144.2 |
| XC_2203 | nucleoside diphosphate kinase | 1.72 | 15.2 |
| XC_0001 | chromosomal replication initiator | 0.00 | 49.5 |
| XC_0510 | purine biosynthesis protein | 0.00 | 55.6 |
| XC_2660 | DNA gyrase subunit A | 0.00 | 98.9 |
| XC_2664 | DNA ligase | 0.00 | 90.3 |
| XC_2744 | adenylosuccinate lyase | 0.00 | 49.8 |
| XC_2804 | replicative DNA helicase | 0.00 | 52.8 |
| XC_3193 | adenylosuccinate synthetase | 0.00 | 46.1 |
| XC_3442 | diadenosine tetraphosphatase | 0.00 | 35.9 |
| XC_4075 | ribonucleoside-diphosphate reductase beta chain | 0.00 | 39.4 |
| 1. **Protein maintenance and folding** | | | |
| XC_3264 | ATP-dependent Clp protease ATP binding subunit | 28.05 | 47.1 |
| XC_3199 | low molecular weight heat shock protein | 11.13 | 17.7 |
| XC_2762 | DnaJ protein | 8.67 | 40.5 |
| XC_0829 | L-isoaspartate protein carboxylmethyltransferase | 8.48 | 24.1 |
| XC_1721 | heat shock protein G | 7.93 | 70.8 |
| XC_3585 | aminopeptidase A/I | 6.19 | 51.6 |
| XC_0213 | protein-export protein | 5.67 | 18.0 |
| XC_3192 | aminopeptidase N | 4.70 | 95.9 |
| XC_1243 | heat shock protein | 4.58 | 32.1 |
| XC_0659 | protease II | 4.12 | 78.0 |
| XC_3144 | alanyl dipeptidyl peptidase | 3.98 | 84.8 |
| XC_0636 | zinc protease | 3.81 | 103.2 |
| XC_3394 | proline iminopeptidase chain A | 3.81 | 35.4 |
| XC_1088 | ATP-dependent Clp protease subunit | 3.63 | 95.4 |
| XC_3265 | ATP-dependent Clp protease proteolytic subunit | 3.51 | 22.8 |
| XC_1542 | metallopeptidase | 1.92 | 72.7 |
| XC_0676 | aminopeptidase | 1.78 | 73.7 |
| XC_0966 | proline dipeptidase | 0.00 | 48.2 |
| XC_2699 | peptidyl-prolyl cis-trans isomerase | 0.00 | 24.9 |
| 1. **Biosynthesis of small molecules** | | | |
| XC_3612 | glucose-1-phosphate thymidylyltransferase | 4.08 | 32.6 |
| XC_0713 | lipoic acid synthetase | 3.36 | 36.9 |
| XC_3613 | dTDP-glucose-4,6-dehydratase | 2.32 | 38.5 |
| XC_1182 | glutathione synthetase | 1.87 | 34.2 |
| XC_0796 | glutaredoxin-like protein | 1.66 | 29.7 |
| XC_0892 | glutamate-1-semialdehyde 2,1-aminomutase | 0.00 | 45.0 |
| XC_2466 | aspartate 1-decarboxylase precursor | 0.00 | 13.6 |
| 1. **Central intermediary metabolism** | | | |
| XC_3609 | phosphomannose isomerase/GDP-mannose pyrophosphorylase | 31.22 | 50.9 |
| XC_3482 | adenosylhomocysteinase | 25.23 | 52.6 |
| XC_2733 | UDP-glucose dehydrogenase | 17.64 | 48.7 |
| XC_1952 | phosphoenolpyruvate synthase | 15.90 | 86.2 |
| XC_2477 | xylose isomerase | 13.90 | 48.8 |
| XC_0821 | NADP-dependent malic enzyme | 11.99 | 84.5 |
| XC_3471 | methionine adenosyltransferase | 6.95 | 43.7 |
| XC_0929 | transketolase 1 | 6.79 | 72.6 |
| XC_2867 | uridylate kinase | 6.54 | 25.7 |
| XC_1930 | UTP-glucose-1-phosphate uridylyltransferase | 5.84 | 32.1 |
| XC_2533 | 2-dehydro-3-deoxyphosphooctonate aldolase | 4.61 | 29.7 |
| XC_3400 | transaldolase B | 4.48 | 34.6 |
| XC_1973 | KDPG and KHG aldolase | 4.22 | 22.8 |
| XC_0833 | 3-isopropylmalate dehydratase large subunit | 3.77 | 51.4 |
| XC_3215 | carboxyphosphonoenolpyruvate phosphonomutase | 3.38 | 31.8 |
| XC_0841 | ketol-acid reductoisomerase | 2.17 | 36.0 |
| XC_3626 | UDP-glucose 4-epimerase | 2.02 | 33.8 |
| XC_0469 | D-ribulose-5-phosphate 3-epimerase | 1.84 | 24.9 |
| XC_0370 | glycerol kinase | 1.63 | 55.2 |
| XC_0122 | 2-keto-3-deoxygluconate kinase | 1.61 | 36.2 |
| XC_0753 | glucosamine-fructose-6-phosphate aminotransferase | 0.00 | 34.7 |
| XC_0851 | inorganic pyrophosphatase | 0.00 | 19.7 |
| XC_1308 | HPr kinase/phosphatase | 0.00 | 35.1 |
| XC_2329 | aconitase | 0.00 | 99.2 |
| XC_3608 | phosphoglucomutase; phosphomannomutase | 0.00 | 49.1 |
| 1. **Degradative enzymes** | | | |
| XC_1609 | polynucleotide phosphorylase | 66.49 | 75.6 |
| XC_2763 | DnaK protein | 26.54 | 68.8 |
| XC_2206 | 3-ketoacyl-CoA thiolase | 7.44 | 41.9 |
| XC_2979 | enoyl-CoA hydratase | 4.87 | 28.2 |
| XC_0256 | acyl-CoA dehydrogenase | 4.11 | 41.5 |
| XC_2655 | histidine ammonia-lyase | 4.05 | 53.1 |
| XC_2941 | leucine dehydrogenase | 2.12 | 39.7 |
| XC_2654 | atrazine chlorohydrolase | 1.74 | 48.7 |
| XC_3642 | fumarylacetoacetate hydrolase | 1.68 | 35.3 |
| XC_0450 | 4-hydroxyphenylpyruvate dioxygenase | 0.00 | 39.9 |
| XC_2324 | c-di-GMP phosphodiesterase A | 0.00 | 80.0 |
| XC_4207 | endo-1,4-beta-xylanase A | 0.00 | 42.2 |
| 1. **Pathogenicity, virulence, and adaptation** | | | |
| XC_3011 | HrpB1 protein | 4.06 | 15.9 |
| XC_3003 | HrcC protein | 0.00 | 64.0 |
| 1. **Regulatory functions** | | | |
| XC_2506 | carbon storage regulator | 9.46 | 7.7 |
| XC_4281 | phage-related regulatory protein cII | 4.29 | 37.1 |
| XC_1118 | transcriptional activator ampR family | 0.00 | 31.4 |
| 1. **ORFs with undefined category** | | | |
| XC_3991 | hydrolase | 3.87 | 26.8 |
| XC_3631 | kinase | 2.16 | 84.9 |
| XC_1787 | ATP-binding protein | 1.68 | 36.0 |
| 1. **Hypothetical proteins** | | | |
| XC_0215 | conserved hypothetical protein | 37.64 | 21.8 |
| XC_1758 | conserved hypothetical protein | 30.78 | 39.6 |
| XC_0344 | conserved hypothetical protein | 10.13 | 33.3 |
| XC_1724 | conserved hypothetical protein | 8.94 | 17.0 |
| XC_1751 | conserved hypothetical protein | 5.98 | 185.9 |
| XC_0723 | conserved hypothetical protein | 5.55 | 13.3 |
| XC_3269 | conserved hypothetical protein | 4.96 | 14.5 |
| XC_1820 | conserved hypothetical protein | 4.36 | 22.5 |
| XC_2747 | conserved hypothetical protein | 2.30 | 57.2 |
| XC_0119 | conserved hypothetical protein | 2.02 | 43.9 |
| XC_2412 | hypothetical protein XC_2412 | 1.96 | 40.1 |
| XC_2632 | conserved hypothetical protein | 1.93 | 28.4 |
| XC_2492 | conserved hypothetical protein | 1.88 | 17.5 |
| XC_0190 | conserved hypothetical protein | 1.85 | 75.5 |
| XC_1616 | conserved hypothetical protein | 1.83 | 11.9 |
| XC_3114 | conserved hypothetical protein | 1.71 | 43.3 |
| XC_3147 | conserved hypothetical protein | 1.71 | 9.9 |
| XC_0221 | conserved hypothetical protein | 0.00 | 11.0 |
| XC_1713 | conserved hypothetical protein | 0.00 | 155.8 |
| XC_2415 | conserved hypothetical protein | 0.00 | 11.2 |
| XC_2418 | hypothetical protein XC_2418 | 0.00 | 18.4 |
| XC_2455 | conserved hypothetical protein | 0.00 | 45.2 |
| XC_2550 | conserved hypothetical protein | 0.00 | 46.4 |
| XC_2689 | conserved hypothetical protein | 0.00 | 83.0 |
| XC_2810 | conserved hypothetical protein | 0.00 | 27.2 |
| XC_3389 | conserved hypothetical protein | 0.00 | 80.4 |
| XC_3831 | conserved hypothetical protein | 0.00 | 7.6 |
| XC_4145 | conserved hypothetical protein | 0.00 | 42.4 |
